# Diagnostic accuracy of intraoperative methods for margin assessment in breast cancer surgery: A systematic review & meta-analysis

**DOI:** 10.1016/j.breast.2024.103749

**Published:** 2024-05-10

**Authors:** Gavin P. Dowling, Cian M. Hehir, Gordon R. Daly, Sandra Hembrecht, Stephen Keelan, Katie Giblin, Maen M. Alrawashdeh, Fiona Boland, Arnold D.K. Hill

**Affiliations:** a Department of Surgery, Royal College of Surgeons in Ireland (RCSI), University of Medicine and Health Sciences, Dublin, Ireland; b Department of Surgery, Beaumont Hospital, Dublin, Ireland; c Data Science Centre, RCSI University of Medicine and Health Sciences, Dublin, Ireland

**Keywords:** Breast conserving surgery, Margin, Breast cancer, Breast surgery

## Abstract

**Purpose:**

There are a wide variety of intraoperative techniques available in breast surgery to achieve low rates for positive margins of excision. The objective of this systematic review was to determine the pooled diagnostic accuracy of intraoperative breast margin assessment techniques that have been evaluated in clinical practice.

**Methods:**

This study was performed in accordance with PRISMA guidelines. A systematic search of the literature was conducted to identify studies assessing the diagnostic accuracy of intraoperative margin assessment techniques. Only clinical studies with raw diagnostic accuracy data as compared with final permanent section histopathology were included in the meta-analysis. A bivariate model for diagnostic meta-analysis was used to determine overall pooled sensitivity and specificity.

**Results:**

Sixty-one studies were eligible for inclusion in this systematic review and meta-analysis. Cytology demonstrated the best diagnostic accuracy, with pooled sensitivity of 0.92 (95 % CI 0.77–0.98) and a pooled specificity of 0.95 (95 % CI 0.90–0.97). The findings also indicate good diagnostic accuracy for optical spectroscopy, with a pooled sensitivity of 0.86 (95 % CI 0.76–0.93) and a pooled specificity of 0.92 (95 % CI 0.82–0.97).

**Conclusion:**

Pooled data indicate that optical spectroscopy, cytology and frozen section have the greatest diagnostic accuracy of currently available intraoperative margin assessment techniques. However, long turnaround time for results and their resource intensive nature has prevented widespread adoption of these methods. The aim of emerging technologies is to compete with the diagnostic accuracy of these established techniques, while improving speed and usability.

## Highlights

• An estimated 20 % of patients who undergo breast conserving surgery will require re-operation for positive or close margins.
• Intraoperative margin assessment techniques identify positive margins during primary surgery, avoiding a second operation.
• Available techniques differ in terms of diagnostic accuracy, turnaround times, cost and practicality.
• Cytology, frozen section & optical spectroscopy demonstrated the best diagnostic accuracy of currently available techniques.

## Abbreviations

CDP: cancer diagnostic probe
CRR: cavity re-excision rate
CTS: ‘click-to-sense’ assay
CYT: cytology
FN: false negative
FP: false positive
FS: frozen section
IBTR: ipsilateral breast tumour recurrence
IOMA: intraoperative margin assessment
IOMRI: intraoperative MRI
IOUS: intraoperative ultrasound
MCT: micro-CT
MP: MarginProbe
NPPV: negative predictive value
OPT: optical spectroscopy
PMR: positive margin rate
PPV: positive predictive value
REIMS: rapid evaporative ionisation mass spectrometry
ROC: receiver operating characteristic
ROR: re-operation rate
SR: specimen radiography
TAT: turnaround time
TN: true negative
TP: true positive
WBI: whole breast irradiation

## 1 Introduction

Breast cancer is the most common cancer in women worldwide [1]. Most breast cancer patients present with early-stage disease, making them suitable candidates for breast-conserving surgery (BCS) [2]. However, an estimated 20 % of patients who undergo BCS require an additional operation for positive or close margins [[3], [4], [5]]. Positive margins are associated with significantly higher local recurrence rates [6,7]. Therefore, achieving adequate margins of excision is a crucial component of breast cancer surgery. Re-operation for positive margins not only has physical consequences, such as delayed adjuvant therapy and impaired cosmetic outcome, but also has psychological and economic repercussions. Given the high rates of re-excision following BCS, there has been significant research in the development of an accurate intraoperative margin assessment (IOMA) method. The purpose of IOMA tools is to identify positive margins during the primary surgery, facilitating further excision during the procedure and thus avoiding a second operation. Breast surgeons have numerous intraoperative techniques available to them, however, there is great variety in the evidence and practicality of these. Currently established IOMA techniques include pathological techniques such as frozen section (FS) and cytology (CYT); and imaging techniques such as specimen radiography (SR) and intraoperative ultrasound (IOUS). To address specific limitations associated with these methods, innovative IOMA tools have emerged; such as optical spectroscopy (OPT), micro-CT (MCT) and MarginProbe (MP). In recent years, there has been extensive research in the development and validation of these novel IOMA techniques for BCS. These emerging technologies aim to challenge the diagnostic accuracy of the currently established IOMA techniques, while improving speed, cost and practicality. This systematic review and meta-analysis aims to evaluate the pooled diagnostic accuracy of IOMA methods, both established and novel, that have been investigated in clinical practice.

## 2 Methods

This systematic review and meta-analysis was reported in accordance with the Preferred Reporting Items for Systematic Reviews and Meta-Analyses (PRISMA) guidelines. Local institutional ethical approval was not required as all data used in this analysis were obtained from a previously published resource. All authors contributed to formulating the study protocol and it was then registered with the International Prospective Register of Systematic Reviews (PROSPERO Registration ID: CRD42022375035).

### 2.1 Search strategy

An electronic search was performed of the PubMed Medline, EMBASE, Cochrane and Scopus databases on November 10, 2022 for relevant studies that would be suitable for inclusion in this study. This search was per-formed by two independent reviewers (GPD & CH), using a pre-determined search strategy. The search was performed of all fields and included the search terms: (‘breast cancer’) AND (‘intraoperative’) AND (‘margin’) linked using the Boolean operator ‘AND.’ All study designs were included. Duplicate studies were manually removed. All titles and abstracts were initially screened, and studies deemed appropriate had their full texts reviewed. These studies were reviewed to ensure inclusion criteria were met for the primary outcome, with discordances in opinion arbitrated through consultation with a third author (GRD).

### 2.2 Inclusion criteria

Studies that reported margin assessment data from 1 or more intraoperative margin assessment technique used during breast surgery for invasive or in situ breast cancer were eligible. Only studies that contained sensitivity and specificity data compared with permanent section histopathology or in whom sensitivity and specificity data could be calculated from the raw data were included. Only studies written in English were included. Included studies were not restricted based on year of publication.

### 2.3 Exclusion criteria

Studies that did not report sensitivity and/or specificity data as compared with permanent section histopathology were excluded, however, data regarding positive predictive values (PPVs), negative predictive values (NPVs) and overall accuracy were not mandatory (these were calculated from the raw data where possible). Studies not written in English were excluded. Abstracts, conference articles, case studies, reviews and meta-analysis were excluded.

### 2.4 Data extraction and quality assessment

Two independent reviewers (GPD and CMH) extracted the following data using a pre-defined electronic spreadsheet: (1) the first author; (2) year of publication; (3) study design; (4) number of patients or samples; (5) mean age of patients; (6) diagnostic accuracy raw data—false negative (FN), false positive (FP), true negative (TN), true positive (TP); (7) percentages of sensitivity, specificity, PPV, NPV, diagnostic accuracy; (8) cavity re-excision rates (CRRs); (9) positive margin rates (PMRs); (10) re-operation rates (RORs); and (11) turn-around time for results. Quality assessment was performed using the QUADAS-2 tool (Supplementary Figs. 8 and 9), designed for evaluating risk of bias in diagnostic accuracy studies [8].

### 2.5 Statistical analysis

Stata version 17 (StataCorp College Station, Texas, USA), particularly the metandi command and metadta, were used for all statistical analyses [9,10]. The number of true positives, false positives, true negatives and false negatives and type of technique were extracted from each study. The number of true positives, false positives, true negatives and false negatives and type of technique were extracted from each study. The bivariate random effects model was applied to estimate summary estimates of sensitivity and specificity and their corresponding 95 % confidence intervals for each technique type. This approach was applied as it preserved the two-dimensional nature of the original data and took into account both study size and heterogeneity beyond chance between studies [11]. Sensitivity referred to the proportion of positive margins correctly classified as positive. Specificity was the proportion negative margins correctly classified negative.

Individual and summary estimates of sensitivity and specificity for the studies investigating each technique were plotted in a receiver operating characteristic (ROC) graph, plotting the rules sensitivity (true positive) on the y axis against 1-specificity (false negative) on the x axis. The 95 % confidence region and 95 % prediction region around the pooled estimates were included to illustrate the precision with which the pooled values were estimated (confidence ellipse around the mean value) and to illustrate the amount of between study variation (prediction ellipse).

Heterogeneity was evaluated visually using the summary ROC plots and statistically by using the variance of logit transformed sensitivity and specificity, with smaller values indicating less heterogeneity among studies. We performed meta-analysis for techniques SR, OPT, CYT, IOUS, FS MCT and MP. However, we acknowledge that there were a very small number of studies in relation to MCT and MP, thus results should be interpreted with caution.

## 3 Results

### 3.1 Literature search

**Fig. 1:**
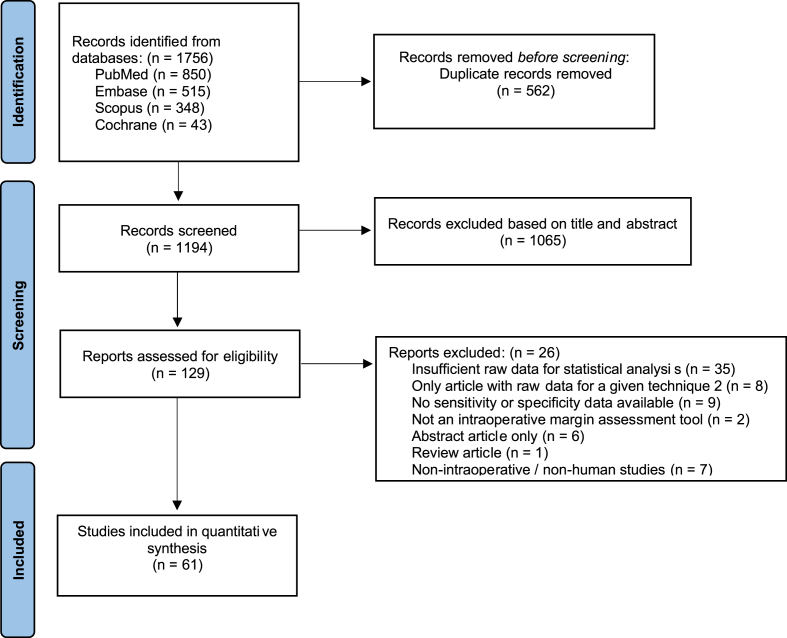
PRISMA flow diagram.

The systematic search strategy identified a total of 1756 studies, of which 562 duplicate studies were manually removed. The remaining 1194 titles and abstracts were screened for relevance, of which 129 studies had their full texts assessed for eligibility. Raw diagnostic accuracy data were unavailable in 35 papers, but were available in 69 papers. To enable meta-analysis, at least 2 studies were required per IOMA group, therefore 8 studies were excluded, as they were the only study for the given technique. Four studies contributed data to 2 IOMA techniques [[12], [13], [14], [15]]. This resulted in a total of 61 studies included for the final analysis, of 7 IOMA techniques (Fig. 1). Quality assessment was performed for each study using the QUADAS-2 tool (Supplementary Figs. 8 and 9).

### 3.2 Study characteristics

Overall, 61 studies were included, and all of these contained sensitivity and specificity percentage data, as well as sufficient raw data to enable meta-analysis. Results are detailed for the 61 studies included in the meta-analysis in Table 1. Forty papers were prospective studies and 21 were retrospective. The studies were published between 1990 and 2022. Mean or median age was available in 39 studies and ranged between 44.9 and 66 years. Distances defined for positive margins varied from 1 mm to 5 mm, with a mode of 2 mm. CRRs were performed within the same operation and PMRs and RORs were performed at an additional operation. Turnaround time for results, when reported, are also listed in Table 1. Reported or calculable percentage sensitivity, specificity, PPV, NPV and overall diagnostic accuracy for each study are listed in Table 2.

**Table 1:** Characteristics of studies included in meta-analysis.

Tech	Author	Year	Study design	Pt	Res	Mar	Ind	M Dist	Age	CRR	PMR	ROR	Time
SR	Lin et al. [45]	2020	Retrospective	202	205		1	5		12.7	18	1	
	Bathla et al. [46]	2011	Retrospective	99	102		1	1	58.6	28.4	17.6	14.7	
	Baù et al. [47]	2020	Prospective	18	18								
	Chagpar et al. [48]	2015	Prospective	90			1	1	60	28.9	30	10	
	Chand et al. [49]	2019	Prospective	30	30	180	2	1	55.67	30		0	
	Ciccarelli et al. [50]	2007	Retrospective	102			2	2		31.4	22.5	20	
	Coombs et al. [51]	2006	Retrospective	101	52		1	5	58.2		19.7	9.3	
	Funk et al. [52]	2019	Retrospective	470	470	2820	1	1	60.2	61.9	7.6	21.7	
	Graham et al. [53]	1994	Prospective		119		2	1					
	Hisada et al. [54]	2016	Retrospective	174					54.7	13.8	20		
	Kulkarni et al. [55]	2021	Prospective	118		708	1	0	62		3.4		
	McCormick et al. [56]	2004	Retrospective	93			1			18		5	15
	Miller et al. [57]	2016	Prospective RCT	36			1		59				
	Park et al. [58]	2019	Retrospective	99		594	1	2	60.2		6	10.1	
	Pop et al. [12]	2018	Prospective	83			1	2		30		9	
	Prueksadee et al. [59]	2009	Retrospective	12			1	2	59.3	50	25		
	Saarela et al. [60]	2001	Prospective	64	66		2	0	55	74.2	16.7		
	Schaefgen et al. [61]	2021	Retrospective	174	174	1044	1	1	51.4	54.6	9.8	9.2	
	Stachs et al. [62]	2022	Prospective RCT	117			1,4	1–2	61.2	35	29.1	27.4	
	Weber et al. [13]	2008	Retrospective		35		1	1	57.5		42.9	37.1	
OPT	Brown et al. [63]	2010	Prospective	57		55	1	2				20	
	Keller et al. [64]	2010	Prospective	40		179	3	1					
	Nguyen et al. [65]	2009	Prospective	20	20	210	1	2	66				
	Schmidt et al. [66]	2019	Prospective	50	185		1,4	0	61	14	14		
	Zhu et al. [67]	2021	Prospective	41			3						
MP	Hoffman et al. [68]	2022	Prospective	48	51	302	1	1	64				
	Karni et al. [69]	2007	Prospective	57	57	314	1	1		15.8	38.6	7	
	LeeVan et al. [70]	2020	Prospective	60		360	1	1	63.5	30	13.3	6.6	
MCT	McClatchy et al. [71]	2018	Prospective	32	32		1	0					
	Qiu et al. [35]	2018	Prospective	30	30		1	0	62				13.5
	Tang et al. [36]	2013	Prospective	6		25	1	2	55			10	
IOUS	Kumar et al. [15]	2021	Prospective	62									
	Londero et al. [72]	2010	Prospective		46	184	1	2	53				3-6.
	Mesurolle et al. [73]	2006	Retrospective		81		1	2	59.1		17.4		3-6.
	Moschetta et al. [74]	2015	Prospective	132			1	2	51				
	Perera et al. [75]	2020	Prospective	95	99	384	2	0		5.3	9.5	2.1	3
	Pop et al. [12]	2018	Prospective	83			1	2		30		9	
	Ramos et al. [76]	2013	Prospective	223	225		1	2	59.5	45.7		4	
FS	Caruso et al. [77]	2011	Retrospective	50	52		1	2		10	10		20
	Ikeda et al. [78]	1997	Retrospective	54	56		1	0	44.9	35.7	12.5	10.7	
	Jorns et al. [79]	2014	Prospective	46			5	2	57.4	23.9	39.1	19.6	22
	Kikuyama et al. [80]	2015	Prospective	220		763	1		51.3				
	Kim et al. [81]	2016	Retrospective	25	29		4	1	53	12	12	0	
	Ko et al. [82]	2017	Prospective	509			3	0	50	12.6	7.2	6.3	
	Kumar et al. [15]	2021	Prospective	62						25.8		0	
	Mahadevappa et al. [14]	2017	Prospective	62			3						
	Noguchi et al. [83]	1995	Prospective	95	100		1			35	24		
	Nowikiewicz et al. [84]	2019	Retrospective	505			1		58.7		14.3		15
	Olson et al. [85]	2007	Retrospective	290	292	1404	1		57.2	24.1		11.4	25
	Osako et al. [86]	2015	Retrospective	1029	1327		1	5		30.3	30.3	0.1	50
	Rusby et al. [87]	2008	Prospective	115		557	1	5	49.5	4.4	7	2.6	20
	Weber et al. [13]	2008	Retrospective		80		1	1	59.6		22.5	12.5	
CYT	Bakhshandeh et al. [88]	2007	Retrospective		100	510	1						20
	Blair et al. [89]	2007	Prospective	20	20	120	1						
	Cox et al. [90]	1991	Prospective	111	111		1		58.4				15
	Creager et al. [91]	2002	Retrospective	137	141	758	1	2	58				20
	D'Halluin et al. [92]	2009	Prospective	396	400		1	2	58.6	38.3		13.3	10
	Ku et al. [93]	1991	Prospective		87		1						15
	Mahadevappa et al. [14]	2017	Prospective	62			3						
	Muttalib et al. [94]	2004	Prospective	26	27		1	1			22.2		22.5
	Sumiyoshi et al. [95]	2010	Prospective	160			1		58.1				
	Tamanuki et al. [96]	2020	Retrospective	522			1	0	62				
	Tohnosu et al. [97]	1998	Prospective	50		200	1	5	52.9				
	Valdes et al. [98]	2007	Prospective	12		72	6			23		33.3	15
	Valdes et al. [99]	2007	Prospective	30		68	5						15

Tech, technique; SR, Specimen Radiography; OPT, Optical Spectroscopy; CYT, Cytology; IOUS, Intraoperative Ultrasound; FS, Frozen Section; MP, Margin Probe; MCT, Micro Computerised Topography; Pt, number of patients; Res, number of resections/specimens; Mar, number of margins; Ind, indication (1: BCS for BC; 2: BCS for impalpable BC; 3: BCS or mastectomy for BC; 4: BCS for DCIS; 5: Re-excision of BC after positive margins; 6: BCS for ILC); M Dist, positive margin distance in mm; CRR, cavity re-excision rate; PMR, positive margin rate; ROR, re-operation rate.

**Table 2:** Raw diagnostic accuracy data of studies included in meta-analysis.

Tech	Author	TP	FP	TN	FN	Total	Sensitivity	Specificity	PPV	NPV	Accuracy
SR	Lin et al. [45]	24	10	158	13	205	64.9	94.1	70.6	92.4	88.8
	Bathla et al. [46]	24	5	56	102	187	58.5	91.8	82.8	76.7	78.4
	Baù et al. [47]	2	0	15	1	18	66.7	100	100	93.8	94.4
	Chagpar et al. [48]	12	12	44	90	158	41.2	78.6	53.9	68.8	64.4
	Chand et al. [49]	4	2	22	2	30	66.7	91.7	66.7	91.7	86.7
	Ciccarelli et al. [50]	25	9	55	102	191	65.8	85.9	73.5	80.9	41.8
	Coombs et al. [51]	12	4	25	52	93	52.2	86.2	75	69.4	39.8
	Funk et al. [52]	114	331	2179	196	2820	36.8	86.8	25.6	91.8	81.3
	Graham et al. [53]	62	1	18	119	200	62	95	98	32	67.2
	Hisada et al. [54]	6	6	106	23	141	20.7	94.6	50	82.2	79.4
	Kulkarni et al. [55]	23	123	538	24	708	48.9	81.4	15.8	95.7	79.2
	McCormick et al. [56]	6	10	72	93	181	54.6	87.8	37.5	93.5	83.9
	Miller et al. [57]	2	2	16	2	22	50	88.9	50	88.9	81.8
	Park et al. [58]	14	61	24	0	99	100	28.2	18.7	100	38.4
	Pop et al. [12]	4	11	63	5	83	44.4	85.1	26.7	92.7	80.7
	Prueksadee et al. [59]	3	3	1	12	19	37.5	25	50	16.7	33.3
	Saarela et al. [60]	9	8	31	66	114	33	79	53	63	61
	Schaefgen et al. [61]	13	62	87	12	174	52	58.4	17.3	87.9	57.5
	Stachs et al. [62]	34	16	67	0	117	70	56.7	54.7	71.7	62.4
	Weber et al. [13]	12	6	9	35	62	60	60	66.7	52.9	60
OPT	Brown et al. [63]	27	7	14	7	55	79.4	66.7	79.4	66.7	74.6
	Keller et al. [64]	29	6	139	5	179	85.3	95.9	82.9	96.5	94
	Nguyen et al. [65]	9	2	9	0	20	100	81.8	81.8	100	90
	Schmidt et al. [66]	11	1	32	6	50	64.7	97	91.7	84.2	86
	Zhu et al. [67]	222	26	454	18	720	92.5	94.6	89.5	96.2	93.9
MP	Hoffman et al. [68]	3	97	192	10	302	23.1	66.4	3	95.1	64.6
	Karni et al. [69]	30	88	184	12	314	71.4	67.7	25.4	93.9	68.2
	LeeVan et al. [70]	17	32	10	1	60	94.4	23.8	34.7	90.9	45
MCT	McClatchy et al. [71]	3	9	18	2	32	60	66.7	25	90	65.6
	Qiu et al. [35]	5	0	20	4	29	55.6	100	100	83.3	86.2
	Tang et al. [36]	5	1	18	1	25	83.3	94.7	83.3	94.7	92
IOUS	Kumar et al. [15]	16	0	46	0	62	100	100	100	100	100
	Londero et al. [72]	8	24	132	20	184	28.6	84.6	25	86.8	76.1
	Mesurolle et al. [73]	30	8	33	10	81	75	80.5	79	76.7	77.8
	Moschetta et al. [74]	16	6	90	20	132	44.4	93.8	72.7	81.8	80.3
	Perera et al. [75]	5	26	349	4	384	55.6	93.1	16.1	98.9	92.2
	Pop et al. [12]	8	25	49	1	83	88.9	66.2	24.2	98	68.7
	Ramos et al. [76]	24	79	116	6	225	80	59.5	95.1	23.3	62.2
FS	Caruso et al. [77]	5	3	44	1	53	83	93	62	97	94
	Ikeda et al. [78]	17	4	34	1	56	94.4	89.5	81	97.1	91.1
	Jorns et al. [79]	12	0	28	6	46	66.7	100	100	82.4	87
	Kikuyama et al. [80]	287	18	440	18	763	94.1	96.1	94.1	96.1	95.3
	Kim et al. [81]	3	1	23	2	29	60	95.8	75	92	89.7
	Ko et al. [82]	120	1	338	24	483	83.3	99.7	99.2	93.4	94.8
	Kumar et al. [15]	10	0	46	6	62	62.5	100	100	88.5	90.3
	Mahadevappa et al. [14]	33	1	28	0	62	100	96.6	97.1	100	98.4
	Noguchi et al. [83]	23	12	64	1	100	95.8	84.2	65.7	98.5	87
	Nowikiewicz et al. [84]	4	0	429	72	505	5.3	100	100	85.6	85.7
	Olson et al. [85]	57	5	1228	21	1311	73.1	99.6	91.9	98.3	98
	Osako et al. [86]	259	53	955	60	1327	81.2	94.7	83	94.1	91.5
	Rusby et al. [87]	39	15	495	8	557	83	97	72.2	98.4	96
	Weber et al. [13]	32	5	35	8	80	80	87.5	86.5	81.4	83.8
CYT	Bakhshandeh et al. [88]	30	7	472	1	510	97	99	81.1	99.8	98.4
	Blair et al. [89]	3	0	115	1	119	75	100	100	99.1	99.2
	Cox et al. [90]	22	3	86	0	111	100	96.6	88	100	97.3
	Creager et al. [91]	12	18	104	3	137	80	85.3	40	97.2	85
	D'Halluin et al. [92]	71	26	304	9	410	88.6	92.2	73.6	97	91.5
	Ku et al. [93]	17	2	68	0	87	100	97.1	89.5	100	97.7
	Mahadevappa et al. [14]	33	1	27	0	61	100	96.4	97.1	100	98.4
	Muttalib et al. [94]	6	6	15	0	27	100	71.4	50	100	77.8
	Sumiyoshi et al. [95]	14	4	136	6	160	70	97.1	77.8	95.8	93.8
	Tamanuki et al. [96]	78	58	375	11	522	87.6	86.6	57.4	97.2	86.8
	Tohnosu et al. [97]	27	16	156	1	200	96.4	90.7	62.8	99.4	91.5
	Valdes et al. [98]	1	1	59	11	72	8.3	98.3	50	84.3	83.3
	Valdes et al. [99]	3	11	53	1	68	75	82.8	21.4	98.2	82.4

Tech, technique; SR, Specimen Radiography; OPT, Optical Spectroscopy; CYT, Cytology; IOUS, Intraoperative Ultrasound; FS, Frozen Section; MP, Margin Probe; MCT, Micro Computerised Topography; TP, true positive; FP, false positive; TN, true negative; FN, false negative; PPV, positive predictive value; NPV, negative predictive value; Accuracy, diagnostic accuracy.

### 3.3 Meta-analysis

**Fig. 2:**
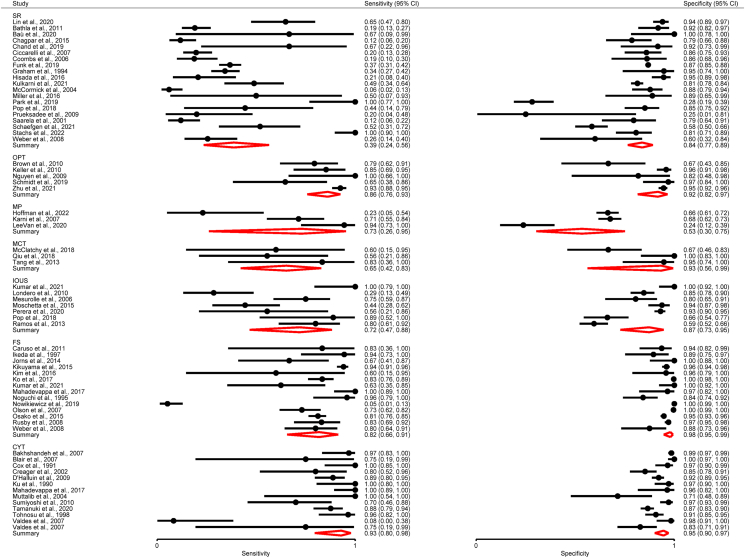
Pooled meta-analysis forest plot for each IOMA technique, displaying sensitivity and specificity data for all studies included and the pooled estimate.

The pooled sensitivity, specificity and the respective variance of the logit transformed sensitivity and specificity for each technique type in the meta-analysis are displayed in Table 3. The forest plot can be seen in Fig. 2.

**Table 3:** Meta-analysis: summary estimates of sensitivity and specificity for all included studies for each IOMA technique type.

Technique	No. of studies (patients/margins)	Sensitivity (95 % CI)	Variance Logit Sensitivity (95 % CI)	Specificity (95 % CI)	Variance Logit Specificity (95 % CI)
SR	20 (5622)	0.39 (0.24–0.56)	2.32 (1.02–5.27)	0.84 (0.77–0.89)	0.89 (0.40–1.94)
OPT	5 (1024)	0.86 (0.76–0.93)	0.34 (0.04–3.13)	0.92 (0.82–0.97)	0.78 (0.13–4.71)
CYT	13 (2484)	0.92 (0.77–0.98)	3.68 (1.11–12.26)	0.95 (0.90–0.97)	1.21 (0.43–3.42)
IOUS	7 (1151)	0.72 (0.47–0.88)	1.58 (0.33–7.62)	0.87 (0.73–0.95)	1.36 (0.31–5.87)
FS	14 (5434)	0.82 (0.66–0.91)	2.30 (0.96–5.54)	0.98 (0.95–0.99)	2.84 (0.91–8.80)
MP^a^	3 (165)	0.73 (0.26–0.95)	2.68	0.53 (0.30–0.75)	0.67
MCT^a^	3 (68)	0.65 (0.42–0.83)	0	0.93 (0.56–0.99)	2.52

a Only three studies and thus results should be interpreted with caution.

These findings indicate that CYT seems best in terms of diagnostic accuracy, with pooled sensitivity of 0.92 (95 % CI 0.77–0.98) and a pooled specificity of 0.95 (95 % CI 0.90–0.97). These findings also indicate good diagnostic accuracy for OPT, with a pooled sensitivity of 0.86 (95 % CI 0.76–0.93) and a pooled specificity of 0.92 (95 % CI 0.82–0.97). These results demonstrate limited diagnostic accuracy for SR. However, the results indicate SR is better at ruling out rather than ruling individuals, with a higher pooled specificity (0.84, 95 % CI 0.77–0.89) compared to sensitivity (0.39, 95 % CI 0.24–0.56). These findings show IOUS to be a superior imaging method for IOMA to SR, with both a higher pooled sensitivity (0.72, 95 % CI 0.47–0.88) and specificity (0.87, 95 % CI 0.73–0.95). Meta-analysis of the 14 studies investigating FS demonstrated the highest pooled specificity (0.98, 95 % CI 0.95–0.99), however limited pooled sensitivity was observed (0.82, 95 % CI 0.66–0.91). MP and MCT both demonstrated limited sensitivity and specificity as IOMA tools, with the exception of the high specificity of MCT (0.93, 95 % CI 0.56–0.99), however these results must be interpreted with due to the limited number of studies available for meta-analysis for each method.

Individual and summary estimates of sensitivity and specificity for all of the studies included in the meta-analysis, the 95 % confidence region and 95 % prediction region are presented for SR, OPT, CYT, IOUS and FS in the summary ROC graphs (Supplementary Figs. 1–5). For SR (Supplementary Fig. 1), OPT (Supplementary Fig. 2) and IOUS (Supplementary Fig. 4), the 95 % confidence regions were broad, reducing the precision of studies in the pooled estimate. The 95 % prediction regions (amount of variation between studies) were also very wide suggesting heterogeneity between studies. This may be, at least in part, explained by the fact that both patient numbers and margin numbers were pooled together in this analysis.

For CYT (Supplementary Fig. 3) and FS (Supplementary Fig. 5) the 95 % confidence region was narrower, and although the 95 % prediction region were narrower compared to the other techniques, they still indicate heterogeneity between studies. The results for MP and MCT are also presented (Supplementary Figs. 6 and 7) and as seen in these plots the results are unreliable.

## 4 Discussion

Breast conserving surgery (BCS) now constitutes the mainstay of treatment, being favoured increasingly over mastectomy [16,17]. However, between 16 and 23.1 % of women treated with BCS undergo re-operation due to incomplete excision or inadequate tumour margins [[18], [19], [20]], with re-operation being associated with undesirable consequences such as delay in adjuvant treatments, inferior cosmetic outcome and most notably; increased risk of local and distant disease recurrence [[21], [22], [23]]. Timely and accurate intraoperative margin assessment (IOMA) may provide a means of reducing re-operation rates which would have a significant impact both with regards to improving patient outcomes and optimising healthcare system productivity and cost-effectiveness [24]. Significant reduction in healthcare costs and re-operation rates have already been demonstrated by IOMA use in some centres [25].

Although the significance of positive tumour margins is widely understood, the definition of negative margins varies significantly within the literature. The studies included in this meta-analysis ranged in definition from ‘no ink on tumour’ to a 5 mm tumour free margin. This disparity has been reflected in the changing guidelines, with most guidelines now recommending “no ink on tumour” as the standard margin for invasive cancer treated with BCS followed by whole breast irradiation (WBI) [7,26]. However, for DCIS the guidelines recommend a 2 mm tumour free margin when treated with BCS and WBI [27]. These guidelines were updated based on results of meta-analyses, which showed a twofold increase in ipsilateral breast tumour recurrence (IBTR) with positive margins in invasive cancer and DCIS (“ink on tumour” and <2 mm, respectively) [28,29].

The present meta-analysis analysed the diagnostic accuracy of a range available IOMA techniques. Many of the techniques analysed showed promising capacity in accurately identifying positive margins. Of those analysed, histopathological means of margin assessment demonstrated superior capabilities in terms of diagnostic accuracy, namely CYT (pooled sensitivity 0.92, pooled specificity 0.95) and FS (pooled sensitivity 0.82, pooled specificity 0.98). Although the diagnostic accuracy demonstrated in both cases is impressive, it must be evaluated within the context of the time and resources required. CYT and FS may add an additional 15 and 30 min respectively to time under anaesthesia [30], and is demanding with regards to requiring timely availability of histopathologists sufficiently experienced in cytopathological assessment in particular. It is likely the resource-intensive nature of these pathological techniques, combined with slow turnaround times, surgical workflow disruptions and considerable financial costs that have prevented them being adopted routinely in clinical practice.

Optical spectroscopy (OPT) is a novel IOMA method that demonstrated impressive diagnostic accuracy (pooled sensitivity 0.86, pooled specificity 0.92) and has promising advantages. It is significantly less demanding from a time and resource perspective [31], with assessment time reported as between 10 and 90 s to obtain an adequate spectroscopic margin profile [32]. Therefore, OPT offers sensitive IOMA within a favourable timeframe, minimising disruption in surgical workflow. However, making real-time surgical decisions based off this spectroscopic profile requires a highly trained and validated classifier, requiring significant training. An ongoing clinical trial is investigating whether artificial intelligence can accurately interpret these optical imaging results [33], with the potential of further improving the turnaround time for results and potentially removing the need for surgeons to be trained in their interpretation.

SR is a well-established radiological IOMA technique and, although it is routinely used in many hospitals for IOMA, showed the lowest diagnostic accuracy of all techniques on meta-analysis (pooled sensitivity 0.39, pooled specificity 0.84). However, SR offers many advantages which may explain its widespread adoption in clinical practice including ease of interpretation by the surgeon, minimal disruption to workflow, fast turnaround times and cost-effectiveness. Other radiological IOMA tools such as IOUS are also time-efficient and demonstrated superior diagnostic accuracy on pooled analysis (pooled sensitivity 0.72, pooled specificity 0.87). Other probe-based tools, such as MP, using radio-frequency spectroscopy, have been shown to significantly reduce the ROR [34], although only demonstrating moderate accuracy on meta-analysis (pooled sensitivity 0.73, pooled specificity 0.53). 3D imaging devices for the operating theatre are currently begin evaluated in an attempt to improve IOMA accuracy. MCT is one such device, and although diagnostic accuracy was unimpressive on pooled analysis (pooled sensitivity 0.65, pooled specificity 0.93), the number of patients included in the analysis was small (n = 68) and thus these results should be interpreted with caution. Individual studies have shown promising results with MCT [35,36], however a major disadvantage of this technique is that currently accurate protocols may require up to 14 min for imaging [36]. Intraoperative-MRI (IOMRI) is also being evaluated as a potential IOMA tool, with limited clinical data to date [37,38].

Many novel IOMA tools are currently being developed, with the aim of addressing some limitations of currently established techniques, as well as improving accuracy. Emerging probe-based tools such as the Cancer Diagnostic Probe (CDP) and the “click-to-sense” assay (CTS), using hypoxia glycolysis and acrolein for tumour cell detection, respectively, have shown promising preliminary results (CDP: sensitivity 100 %, specificity 92.3 %; CTS: sensitivity 93.3 %, specificity 98.3 %) [39,40]. Confocal microscopy is another technology which has shown encouraging preliminary results (sensitivity 91–97 %, specificity 86–93 %) [41,42]. Rapid evaporative ionisation mass spectrometry (REIMS) is an interesting technology which may enable an “intelligent knife” to analyse margins for cancer intraoperatively [43], and is currently being investigated in a clinical trial [44].

This study is subject to a number of limitations. As previously mentioned, positive margin definitions of included studies ranged from ‘no ink on tumour’ to a 5 mm tumour free margin. This variance in margin definition may constitute an inherent limitation of this study, similarly the participation criteria varied between studies. Another considerable source of heterogeneity is the fact that some studies reported sensitivity and specificity results by means of resection specimen or margin number as opposed to patient number. As this is a relatively novel area of interest, the number of studies included was small for some IOMA techniques, in particular MCT and MP, and these results should be interpreted with caution. Finally, although diagnostic accuracy is important, re-excision rates are the primary outcome by which these tools will ultimately be measured and remain the most significant in altering clinical practice.

This meta-analysis generated meaningful appraisal of IOMA means with regards to pooled sensitivity and specificity values. Although diagnostic accuracy is of primary importance, the real-world utility and application of each IOMA means is also affected by; capacity for timely inspection and results, ease of result interpretation, requirement for additional personnel/resources for investigation and/or interpretation and financial viability. Due to the global disparity with regards to available resources within the acute hospital setting, the optimal IOMA means may inevitably differ between healthcare systems.

## Funding

This research did not receive any specific grant from funding agencies in the public, commercial, or not-for-profit sectors.

## Ethics approval and consent to participate

Not applicable.

## Consent for publication

Not applicable.

## Availability of data and materials

The datasets used and/or analysed during the current study are available from the corresponding author on reasonable request.

## CRediT authorship contribution statement

**Gavin P. Dowling:** Writing – review & editing, Writing – original draft, Visualization, Investigation, Formal analysis, Data curation, Conceptualization. **Cian M. Hehir:** Writing – review & editing, Formal analysis, Data curation. **Gordon R. Daly:** Writing – review & editing, Methodology, Data curation. **Sandra Hembrecht:** Writing – review & editing, Supervision, Data curation. **Stephen Keelan:** Writing – review & editing, Supervision, Methodology. **Katie Giblin:** Writing – review & editing, Visualization, Data curation. **Maen M. Alrawashdeh:** Writing – review & editing, Software, Data curation. **Fiona Boland:** Writing – review & editing, Visualization, Software, Methodology, Formal analysis. **Arnold D.K. Hill:** Writing – review & editing, Validation, Supervision, Project administration, Methodology, Investigation, Conceptualization.

## Declaration of competing interest

The authors have no conflicts of interest to declare.
