# Supplementary material for: Diagnostic accuracy of intraoperative methods for margin assessment in breast cancer surgery: A systematic review & meta-analysis
Source: Breast. 2024 May 10;76:103749. doi: 10.1016/j.breast.2024.103749 (PMC11127275; doi:10.1016/j.breast.2024.103749)

**Diagnostic Accuracy of Intraoperative Methods for Margin Assessment in Breast Cancer Surgery: A Systematic review & Meta-analysis**

Gavin P. Dowling^1,2^, Cian M. Hehir^1,2^, Gordon R. Daly^1,2^, Sandra Hembrecht^1,2^, Stephen Keelan^1,2^, Katie Giblin^1,2^, Maen M. Alrawashdeh^1^, Fiona Boland^3^, Arnold D.K. Hill^1,2^

1. Department of Surgery, Royal College of Surgeons in Ireland (RCSI) University of Medicine and Health Sciences, Dublin, Ireland
2. Department of Surgery, Beaumont Hospital, Dublin, Ireland
3. Data Science Centre, RCSI University of Medicine and Health Sciences, Dublin, Ireland

Corresponding author:

Gavin P. Dowling

e-mail: [gavindowling@rcsi.com](mailto:gavindowling@rcsi.com)

ORCID ID: 0000-0002-2267-2022

**Abstract**

**Purpose**

There are a wide variety of intraoperative techniques available in breast surgery to achieve low rates for positive margins of excision. The objective of this systematic review was to determine the pooled diagnostic accuracy of intraoperative breast margin assessment techniques that have been evaluated in clinical practice.

**Methods**

This study was performed in accordance with PRISMA guidelines. A systematic search of the literature was conducted to identify studies assessing the diagnostic accuracy of intraoperative margin techniques. Only clinical studies with raw diagnostic accuracy data as compared with final permanent section histopathology were included in the meta-analysis. A bivariate model for diagnostic meta-analysis was used to determine overall pooled sensitivity and specificity.

**Results**

Sixty-one studies were eligible for inclusion in this systematic review and meta-analysis. Cytology demonstrated the best diagnostic accuracy, with pooled sensitivity of 0.92 (95% CI 0.77-0.98) and a pooled specificity of 0.95 (95% CI 0.90-0.97). The findings also indicate good diagnostic accuracy for optical spectroscopy, with a pooled sensitivity of 0.86 (95% CI 0.76-0.93) and a pooled specificity of 0.92 (95% CI 0.82-0.97).

**Conclusion**

Pooled data indicate that optical spectroscopy, cytology and frozen section have the greatest diagnostic accuracy of currently available intraoperative margin assessment techniques. However, long turnaround time for results and their resource intensive nature has prevented widespread adoption of these methods. The aim of emerging technologies is to compete with the diagnostic accuracy of these established techniques, while improving speed and usability.

**Keywords:** Breast conserving surgery, margin, breast cancer, breast surgery

**Introduction**

Breast cancer is the most common cancer in women worldwide^1^. Most breast cancer patients present with early-stage disease, making them suitable candidates for breast-conserving surgery (BCS)^2^. However, an estimated 20% of patients who undergo BCS require an additional operation for positive or close margins^3-5^. Positive margins are associated with significantly higher local recurrence rates^6,7^. Therefore, achieving adequate margins of excision is a crucial component of breast cancer surgery. Re-operation for positive margins not only has physical consequences, such as delayed adjuvant therapy and impaired cosmetic outcome, but also has psychological and economic repercussions. Given the high rates of re-excision following BCS, there has been significant research in the development of an accurate intraoperative margin assessment (IOMA) method. The purpose of IOMA tools is to identify positive margins during the primary surgery, facilitating further excision during the procedure and thus avoiding a second operation. Breast surgeons have numerous intraoperative techniques available to them, however, there is great variety in the evidence and practicality of these. Currently established IOMA techniques include pathological techniques such as frozen section (FS) and cytology (CYT); and imaging techniques such as specimen radiography (SR) and intraoperative ultrasound (IOUS). To address specific limitations associated with these methods, innovative IOMA tools have emerged; such as optical spectroscopy (OPT), micro-CT (MCT) and MarginProbe (MP). In recent years, there has been extensive research in the development and validation of these novel IOMA techniques for BCS. These emerging technologies aim to challenge the diagnostic accuracy of the currently established IOMA techniques, while improving speed, cost and practicality. This systematic review and meta-analysis aims to evaluate the pooled diagnostic accuracy of IOMA methods, both established and novel, that have been investigated in clinical practice.

**Methods**

This systematic review and meta-analysis was reported in accordance with the Preferred Reporting Items for Systematic Reviews and Meta-Analyses (PRISMA) guidelines. Local institutional ethical approval was not required as all data used in this analysis were obtained from a previously published resource. All authors contributed to formulating the study protocol and it was then registered with the International Prospective Register of Systematic Reviews (PROSPERO Registration ID: CRD42022375035).

**Search Strategy**

An electronic search was performed of the PubMed Medline, EMBASE, Cochrane and Scopus databases on 10 November 2022 for relevant studies that would be suitable for inclusion in this study. This search was per- formed by two independent reviewers (GPD & CH), using a pre-determined search strategy. The search was performed of all fields and included the search terms: (‘breast cancer’) AND (‘intraoperative’) AND (‘margin’) linked using the Boolean operator ‘AND.’ All study designs were included. Duplicate studies were manually removed. All titles and abstracts were initially screened, and studies deemed appropriate had their full texts reviewed. These studies were reviewed to ensure inclusion criteria were met for the primary outcome, with discordances in opinion arbitrated through consultation with a third author (GRD).

**Inclusion criteria**

Studies that reported margin assessment data from 1 or more intraoperative margin assessment technique used during breast surgery for invasive or in situ breast cancer were eligible. Only studies that contained sensitivity and specificity data compared with permanent section histopathology or in whom sensitivity and specificity data could be calculated from the raw data were included. Only studies written in English were included. Included studies were not restricted based on year of publication.

**Exclusion criteria**

Studies that did not report sensitivity and/or specificity data as compared with permanent section histopathology were excluded, however, data regarding positive predictive values (PPVs), negative predictive values (NPVs) and overall accuracy were not mandatory (these were calculated from the raw data where possible). Studies not written in English were excluded. Abstracts, conference articles, case studies, reviews and meta-analysis were excluded.

**Data extraction and quality assessment**

Two independent reviewers (GPD and CMH) extracted the following data using a pre-defined electronic spreadsheet: (1) the first author; (2) year of publication; (3) study design; (4) number of patients or samples; (5) mean age of patients; (6) diagnostic accuracy raw data—false negative (FN), false positive (FP), true negative (TN), true positive (TP); (7) percentages of sensitivity, specificity, PPV, NPV, diagnostic accuracy; (8) cavity re-excision rates (CRRs); (9) positive margin rates (PMRs); (10) re-operation rates (RORs); and (11) turn- around time for results. Quality assessment was performed using the QUADAS-2 tool (Supplementary Figures 8 & 9), designed for evaluating risk of bias in diagnostic accuracy studies^8^.

**Statistical analysis**

Stata version 17 (StataCorp College Station, Texas, USA), particularly the metandi command and metadta, were used for all statistical analyses ^9,10^. The number of true positives, false positives, true negatives and false negatives and type of technique were extracted from each study. The number of true positives, false positives, true negatives and false negatives and type of technique were extracted from each study. The bivariate random effects model was applied to estimate summary estimates of sensitivity and specificity and their corresponding 95% confidence intervals for each technique type. This approach was applied as it preserved the two-dimensional nature of the original data and took into account both study size and heterogeneity beyond chance between studies ^11^. Sensitivity referred to the proportion of positive margins correctly classified as positive. Specificity was the proportion negative margins correctly classified negative.

Individual and summary estimates of sensitivity and specificity for the studies investigating each technique were plotted in a receiver operating characteristic (ROC) graph, plotting the rules sensitivity (true positive) on the y axis against 1-specificity (false negative) on the x axis. The 95% confidence region and 95% prediction region around the pooled estimates were included to illustrate the precision with which the pooled values were estimated (confidence ellipse around the mean value) and to illustrate the amount of between study variation (prediction ellipse).

Heterogeneity was evaluated visually using the summary ROC plots and statistically by using the variance of logit transformed sensitivity and specificity, with smaller values indicating less heterogeneity among studies. We performed meta-analysis for techniques SR, OPT, CYT, IOUS, FS MCT and MP. However, we acknowledge that there were a very small number of studies in relation to MCT and MP, thus results should be interpreted with caution.

**Results**

**Literature search**

The systematic search strategy identified a total of 1756 studies, of which 562 duplicate studies were manually removed. The remaining 1194 titles and abstracts were screened for relevance, of which 129 studies had their full texts assessed for eligibility. Raw diagnostic accuracy data were unavailable in 35 papers, but were available in 69 papers. To enable meta-analysis, at least 2 studies were required per IOMA group, therefore 8 studies were excluded, as they were the only study for the given technique. Four studies contributed data to 2 IOMA techniques^12-15^. This resulted in a total of 61 studies included for the final analysis, of 7 IOMA techniques (Figure 1). Quality assessment was performed for each study using the QUADAS-2 tool (Supplementary Figures 8 & 9).

**Study characteristics**

Overall, 61 studies were included, and all of these contained sensitivity and specificity percentage data, as well as sufficient raw data to enable meta-analysis. Results are detailed for the 61 studies included in the meta-analysis in Table 1. Forty papers were prospective studies and 21 were retrospective. The studies were published between 1990 to 2022. Mean or median age was available in 39 studies and ranged between 44.9 to 66 years. Distances defined for positive margins varied from 1mm to 5mm, with a mode of 2mm. CRRs were performed within the same operation and PMRs and RORs were performed at an additional operation. Turnaround time for results, when reported, are also listed in Table 1. Reported or calculable percentage sensitivity, specificity, PPV, NPV and overall diagnostic accuracy for each study are listed in Table 2.

**Meta-Analysis**

The pooled sensitivity, specificity and the respective variance of the logit transformed sensitivity and specificity for each technique type in the meta-analysis are displayed in Table 3. The forest plot can be seen in Figure 2.

These findings indicate that CYT seems best in terms of diagnostic accuracy, with pooled sensitivity of 0.92 (95% CI 0.77-0.98) and a pooled specificity of 0.95 (95% CI 0.90-0.97). These findings also indicate good diagnostic accuracy for OPT, with a pooled sensitivity of 0.86 (95% CI 0.76-0.93) and a pooled specificity of 0.92 (95% CI 0.82-0.97). These results demonstrate limited diagnostic accuracy for SR. However, the results indicate SR is better at ruling out rather than ruling individuals, with a higher pooled specificity (0.84, 95% CI 0.77-0.89) compared to sensitivity (0.39, 95% CI 0.24-0.56). These findings show IOUS to be a superior imaging method for IOMA to SR, with both a higher pooled sensitivity (0.72, 95% CI 0.47-0.88) and specificity (0.87, 95% CI 0.73-0.95). Meta-analysis of the 14 studies investigating FS demonstrated the highest pooled specificity (0.98, 95% CI 0.95-0.99), however limited pooled sensitivity was observed (0.82, 95% CI 0.66-0.91). MP and MCT both demonstrated limited sensitivity and specificity as IOMA tools, with the exception of the high specificity of MCT (0.93, 95% CI 0.56-0.99), however these results must be interpretated with due to the limited number of studies available for meta-analysis for each method.

Individual and summary estimates of sensitivity and specificity for all of the studies included in the meta-analysis, the 95% confidence region and 95% prediction region are presented for SR, OPT, CYT, IOUS and FS in the summary ROC graphs (Supplementary Figures 1-5). For SR (Supplementary Figure 1), OPT (Supplementary Figure 2) and IOUS (Supplementary Figure 4), the 95% confidence regions were broad, reducing the precision of studies in the pooled estimate. The 95% prediction regions (amount of variation between studies) were also very wide suggesting heterogeneity between studies. This may be, at least in part, explained by the fact that both patient numbers and margin numbers were pooled together in this analysis.

For CYT (Supplementary Figure 3) and FS (Supplementary Figure 5) the 95% confidence region was narrower, and although the 95% prediction region were narrower compared to the other techniques, they still indicate heterogeneity between studies. The results for MP and MCT are also presented (Supplementary Figures 6 & 7) and as seen in these plots the results are unreliable.

**Discussion**

Breast conserving surgery (BCS) now constitutes the mainstay of treatment, being favoured increasingly over mastectomy^16,17^. However, between 16-23.1% of women treated with BCS undergo re-operation due to incomplete excision or inadequate tumour margins^18-20^, with re-operation being associated with undesirable consequences such as delay in adjuvant treatments, inferior cosmetic outcome and most notably; increased risk of local and distant disease recurrence^21-23^. Timely and accurate intraoperative margin assessment (IOMA) may provide a means of reducing re-operation rates which would have a significant impact both with regards to improving patient outcomes and optimising healthcare system productivity and cost-effectiveness^24^. Significant reduction in healthcare costs and re-operation rates have already been demonstrated by IOMA use in some centres^25^.

Although the significance of positive tumour margins is widely understood, the definition of negative margins varies significantly within the literature. The studies included in this meta-analysis ranged in definition from ‘no ink on tumour’ to a 5mm tumour free margin. This disparity has been reflected in the changing guidelines, with most guidelines now recommending “no ink on tumour” as the standard margin for invasive cancer treated with BCS followed by whole breast irradiation (WBI)^7,26^. However, for DCIS the guidelines recommend a 2mm tumour free margin when treated with BCS and WBI^27^. These guidelines were updated based on results of meta-analyses, which showed a twofold increase in ipsilateral breast tumour recurrence (IBTR) with positive margins in invasive cancer and DCIS (“ink on tumour” and <2mm, respectively)^28,29^.

The present meta-analysis analysed the diagnostic accuracy of a range available IOMA techniques. Many of the techniques analysed showed promising capacity in accurately identifying positive margins. Of those analysed, histopathological means of margin assessment demonstrated superior capabilities in terms of diagnostic accuracy, namely CYT (pooled sensitivity 0.92, pooled specificity 0.95) and FS (pooled sensitivity 0.82, pooled specificity 0.98). Although the diagnostic accuracy demonstrated in both cases is impressive, it must be evaluated within the context of the time and resources required. CYT and FS may add an additional 15 and 30 minutes respectively to time under anaesthesia,^30^ and is demanding with regards to requiring timely availability of histopathologists sufficiently experienced in cytopathological assessment in particular. It is likely the resource-intensive nature of these pathological techniques, combined with slow turnaround times, surgical workflow disruptions and considerable financial costs that have prevented them being adopted routinely in clinical practice.

Optical spectroscopy (OPT) is a novel IOMA method that demonstrated impressive diagnostic accuracy (pooled sensitivity 0.86, pooled specificity 0.92) and has promising advantages. It is significantly less demanding from a time and resource perspective^31^, with assessment time reported as between 10-90 seconds to obtain an adequate spectroscopic margin profile^32^. Therefore, OPT offers sensitive IOMA within a favourable timeframe, minimising disruption in surgical workflow. However, making real-time surgical decisions based off this spectroscopic profile requires a highly trained and validated classifier, requiring significant training. An ongoing clinical trial is investigating whether artificial intelligence can accurately interpret these optical imaging results^33^, with the potential of further improving the turnaround time for results and potentially removing the need for surgeons to be trained in their interpretation.

SR is a well-established radiological IOMA technique and, although it is routinely used in many hospitals for IOMA, showed the lowest diagnostic accuracy of all techniques on meta-analysis (pooled sensitivity 0.39, pooled specificity 0.84). However, SR offers many advantages which may explain its widespread adoption in clinical practice including ease of interpretation by the surgeon, minimal disruption to workflow, fast turnaround times and cost-effectiveness. Other radiological IOMA tools such as IOUS are also time-efficient and demonstrated superior diagnostic accuracy on pooled analysis (pooled sensitivity 0.72, pooled specificity 0.87). Other probe-based tools, such as MP, using radio-frequency spectroscopy, have been shown to significantly reduce the ROR^34^, although only demonstrating moderate accuracy on meta-analysis (pooled sensitivity 0.73, pooled specificity 0.53). 3D imaging devices for the operating theatre are currently begin evaluated in an attempt to improve IOMA accuracy. MCT is one such device, and although diagnostic accuracy was unimpressive on pooled analysis (pooled sensitivity 0.65, pooled specificity 0.93), the number of patients included in the analysis was small (n=68) and thus these results should be interpreted with caution. Individual studies have shown promising results with MCT^35,36^, however a major disadvantage of this technique is that currently accurate protocols may require up to 14 minutes for imaging^36^. Intraoperative-MRI (IOMRI) is also being evaluated as a potential IOMA tool, with limited clinical data to date^37,38^.

Many novel IOMA tools are currently being developed, with the aim of addressing some limitations of currently established techniques, as well as improving accuracy. Emerging probe-based tools such as the Cancer Diagnostic Probe (CDP) and the “click-to-sense” assay (CTS), using hypoxia glycolysis and acrolein for tumour cell detection, respectively, have shown promising preliminary results (CDP: sensitivity 100%, specificity 92.3%; CTS: sensitivity 93.3%, specificity 98.3%)^39,40^. Confocal microscopy is another technology which has shown encouraging preliminary results (sensitivity 91-97%, specificity 86-93%)^41,42^. Rapid evaporative ionisation mass spectrometry (REIMS) is an interesting technology which may enable an “intelligent knife” to analyse margins for cancer intraoperatively^43^, and is currently being investigated in a clinical trial^44^.

This study is subject to a number of limitations. As previously mentioned, positive margin definitions of included studies ranged from ‘no ink on tumour’ to a 5mm tumour free margin. This variance in margin definition may constitute an inherent limitation of this study, similarly the participation criteria varied between studies. Another considerable source of heterogeneity is the fact that some studies reported sensitivity and specificity results by means of resection specimen or margin number as opposed to patient number. As this is a relatively novel area of interest, the number of studies included was small for some IOMA techniques, in particular MCT and MP, and these results should be interpreted with caution. Finally, although diagnostic accuracy is important, re-excision rates are the primary outcome by which these tools will ultimately be measured and remain the most significant in altering clinical practice.

This meta-analysis generated meaningful appraisal of IOMA means with regards to pooled sensitivity and specificity values. Although diagnostic accuracy is of primary importance, the real-world utility and application of each IOMA means is also affected by; capacity for timely inspection and results, ease of result interpretation, requirement for additional personnel/resources for investigation and/or interpretation and financial viability. Due to the global disparity with regards to available resources within the acute hospital setting, the optimal IOMA means may inevitably differ between healthcare systems.

**Declarations**

**Funding**

This research did not receive any specific grant from funding agencies in the public, commercial, or not-for-profit sectors.

**Conflict of interest**

The authors have no conflicts of interest to declare.

**Ethics approval and consent to participate**

Not applicable.

**Consent for publication**

Not applicable.

**Availability of data and materials**

The datasets used and/or analyzed during the current study are available from the corresponding author on reasonable request.

**Author contributions**

Conceptualisation; GPD, AKDH, SK. Data curation; GPD, CMH, GRD, SH, KG, MMA. Formal analysis; FB, GPD. Writing of original draft; GPD, CMH, GRD. Review & editing; FB, SH, SK, KG, MMA, ADKH.

**Abbreviations**

CDP – cancer diagnostic probe

CRR – cavity re-excision rate

CTS – ‘click-to-sense’ assay

CYT – cytology

FN – false negative

FP – false positive

FS – frozen section

IBTR – ipsilateral breast tumour recurrence

IOMA – intraoperative margin assessment

IOMRI – intraoperative MRI

IOUS – intraoperative ultrasound

MCT – micro-CT

MP – MarginProbe

NPPV – negative predictive value

OPT – optical spectroscopy

PMR – positive margin rate

PPV – positive predictive value

REIMS - rapid evaporative ionisation mass spectrometry

ROC – receiver operating characteristic

ROR – re-operation rate

SR – specimen radiography

TAT – turnaround time

TN – true negative

TP – true positive

WBI – whole breast irradiation

**Tables and Figures:**

Table 1. Characteristics of studies included in meta-analysis

| Tech | Author | Year | Study design | Pt | Res | Mar | Ind | M Dist | Age | CRR | PMR | ROR | Time |
| --- | --- | --- | --- | --- | --- | --- | --- | --- | --- | --- | --- | --- | --- |
| SR | Lin et al^45^ | 2020 | Retrospective | 202 | 205 |  | 1 | 5 |  | 12.7 | 18 | 1 |  |
|  | Bathla et al^46^ | 2011 | Retrospective | 99 | 102 |  | 1 | 1 | 58.6 | 28.4 | 17.6 | 14.7 |  |
|  | Baù et al^47^ | 2020 | Prospective | 18 | 18 |  |  |  |  |  |  |  |  |
|  | Chagpar et al^48^ | 2015 | Prospective | 90 |  |  | 1 | 1 | 60 | 28.9 | 30 | 10 |  |
|  | Chand et al ^49^ | 2019 | Prospective | 30 | 30 | 180 | 2 | 1 | 55.67 | 30 |  | 0 |  |
|  | Ciccarelli et al ^50^ | 2007 | Retrospective | 102 |  |  | 2 | 2 |  | 31.4 | 22.5 | 20 |  |
|  | Coombs et al^51^ | 2006 | Retrospective | 101 | 52 |  | 1 | 5 | 58.2 |  | 19.7 | 9.3 |  |
|  | Funk et al^52^ | 2019 | Retrospective | 470 | 470 | 2820 | 1 | 1 | 60.2 | 61.9 | 7.6 | 21.7 |  |
|  | Graham et al^53^ | 1994 | Prospective |  | 119 |  | 2 | 1 |  |  |  |  |  |
|  | Hisada et al^54^ | 2016 | Retrospective | 174 |  |  |  |  | 54.7 | 13.8 | 20 |  |  |
|  | Kulkarni et al^55^ | 2021 | Prospective | 118 |  | 708 | 1 | 0 | 62 |  | 3.4 |  |  |
|  | McCormick et al^56^ | 2004 | Retrospective | 93 |  |  | 1 |  |  | 18 |  | 5 | 15 |
|  | Miller et al^57^ | 2016 | Prospective RCT | 36 |  |  | 1 |  | 59 |  |  |  |  |
|  | Park et al^58^ | 2019 | Retrospective | 99 |  | 594 | 1 | 2 | 60.2 |  | 6 | 10.1 |  |
|  | Pop et al^12^ | 2018 | Prospective | 83 |  |  | 1 | 2 |  | 30 |  | 9 |  |
|  | Prueksadee et al^59^ | 2009 | Retrospective | 12 |  |  | 1 | 2 | 59.3 | 50 | 25 |  |  |
|  | Saarela et al^60^ | 2001 | Prospective | 64 | 66 |  | 2 | 0 | 55 | 74.2 | 16.7 |  |  |
|  | Schaefgen et al^61^ | 2021 | Retrospective | 174 | 174 | 1044 | 1 | 1 | 51.4 | 54.6 | 9.8 | 9.2 |  |
|  | Stachs et al^62^ | 2022 | Prospective RCT | 117 |  |  | 1,4 | 1-2 | 61.2 | 35 | 29.1 | 27.4 |  |
|  | Weber et al^13^ | 2008 | Retrospective |  | 35 |  | 1 | 1 | 57.5 |  | 42.9 | 37.1 |  |
| OPT | Brown et al^63^ | 2010 | Prospective | 57 |  | 55 | 1 | 2 |  |  |  | 20 |  |
|  | Keller et al^64^ | 2010 | Prospective | 40 |  | 179 | 3 | 1 |  |  |  |  |  |
|  | Nguyen et al^65^ | 2009 | Prospective | 20 | 20 | 210 | 1 | 2 | 66 |  |  |  |  |
|  | Schmidt et al^66^ | 2019 | Prospective | 50 | 185 |  | 1,4 | 0 | 61 | 14 | 14 |  |  |
|  | Zhu et al^67^ | 2021 | Prospective | 41 |  |  | 3 |  |  |  |  |  |  |
| MP | Hoffman et al^68^ | 2022 | Prospective | 48 | 51 | 302 | 1 | 1 | 64 |  |  |  |  |
|  | Karni et al^69^ | 2007 | Prospective | 57 | 57 | 314 | 1 | 1 |  | 15.8 | 38.6 | 7 |  |
|  | LeeVan et al^70^ | 2020 | Prospective | 60 |  | 360 | 1 | 1 | 63.5 | 30 | 13.3 | 6.6 |  |
| MCT | McClatchy et al ^71^ | 2018 | Prospective | 32 | 32 |  | 1 | 0 |  |  |  |  |  |
|  | Qiu et al^35^ | 2018 | Prospective | 30 | 30 |  | 1 | 0 | 62 |  |  |  | 13.5 |
|  | Tang et al^36^ | 2013 | Prospective | 6 |  | 25 | 1 | 2 | 55 |  |  | 10 |  |
| IOUS | Kumar et al^15^ | 2021 | Prospective | 62 |  |  |  |  |  |  |  |  |  |
|  | Londero et al^72^ | 2010 | Prospective |  | 46 | 184 | 1 | 2 | 53 |  |  |  | 3-6. |
|  | Mesurolle et al^73^ | 2006 | Retrospective |  | 81 |  | 1 | 2 | 59.1 |  | 17.4 |  | 3-6. |
|  | Moschetta et al^74^ | 2015 | Prospective | 132 |  |  | 1 | 2 | 51 |  |  |  |  |
|  | Perera et al ^75^ | 2020 | Prospective | 95 | 99 | 384 | 2 | 0 |  | 5.3 | 9.5 | 2.1 | 3 |
|  | Pop et al ^12^ | 2018 | Prospective | 83 |  |  | 1 | 2 |  | 30 |  | 9 |  |
|  | Ramos et al ^76^ | 2013 | Prospective | 223 | 225 |  | 1 | 2 | 59.5 | 45.7 |  | 4 |  |
| FS | Caruso et al^77^ | 2011 | Retrospective | 50 | 52 |  | 1 | 2 |  | 10 | 10 |  | 20 |
|  | Ikeda et al^78^ | 1997 | Retrospective | 54 | 56 |  | 1 | 0 | 44.9 | 35.7 | 12.5 | 10.7 |  |
|  | Jorns et al^79^ | 2014 | Prospective | 46 |  |  | 5 | 2 | 57.4 | 23.9 | 39.1 | 19.6 | 22 |
|  | Kikuyama et al^80^ | 2015 | Prospective | 220 |  | 763 | 1 |  | 51.3 |  |  |  |  |
|  | Kim et al^81^ | 2016 | Retrospective | 25 | 29 |  | 4 | 1 | 53 | 12 | 12 | 0 |  |
|  | Ko et al^82^ | 2017 | Prospective | 509 |  |  | 3 | 0 | 50 | 12.6 | 7.2 | 6.3 |  |
|  | Kumar et al ^15^ | 2021 | Prospective | 62 |  |  |  |  |  | 25.8 |  | 0 |  |
|  | Mahadevappa et al^14^ | 2017 | Prospective | 62 |  |  | 3 |  |  |  |  |  |  |
|  | Noguchi et al^83^ | 1995 | Prospective | 95 | 100 |  | 1 |  |  | 35 | 24 |  |  |
|  | Nowikiewicz et al^84^ | 2019 | Retrospective | 505 |  |  | 1 |  | 58.7 |  | 14.3 |  | 15 |
|  | Olson et al^85^ | 2007 | Retrospective | 290 | 292 | 1404 | 1 |  | 57.2 | 24.1 |  | 11.4 | 25 |
|  | Osako et al^86^ | 2015 | Retrospective | 1029 | 1327 |  | 1 | 5 |  | 30.3 | 30.3 | 0.1 | 50 |
|  | Rusby et al^87^ | 2008 | Prospective | 115 |  | 557 | 1 | 5 | 49.5 | 4.4 | 7 | 2.6 | 20 |
|  | Weber et al^13^ | 2008 | Retrospective |  | 80 |  | 1 | 1 | 59.6 |  | 22.5 | 12.5 |  |
| CYT | Bakhshandeh et al^88^ | 2007 | Retrospective |  | 100 | 510 | 1 |  |  |  |  |  | 20 |
|  | Blair et al^89^ | 2007 | Prospective | 20 | 20 | 120 | 1 |  |  |  |  |  |  |
|  | Cox et al^90^ | 1991 | Prospective | 111 | 111 |  | 1 |  | 58.4 |  |  |  | 15 |
|  | Creager et al^91^ | 2002 | Retrospective | 137 | 141 | 758 | 1 | 2 | 58 |  |  |  | 20 |
|  | D'Halluin et al^92^ | 2009 | Prospective | 396 | 400 |  | 1 | 2 | 58.6 | 38.3 |  | 13.3 | 10 |
|  | Ku et al^93^ | 1991 | Prospective |  | 87 |  | 1 |  |  |  |  |  | 15 |
|  | Mahadevappa et al^14^ | 2017 | Prospective | 62 |  |  | 3 |  |  |  |  |  |  |
|  | Muttalib et al^94^ | 2004 | Prospective | 26 | 27 |  | 1 | 1 |  |  | 22.2 |  | 22.5 |
|  | Sumiyoshi et al^95^ | 2010 | Prospective | 160 |  |  | 1 |  | 58.1 |  |  |  |  |
|  | Tamanuki et al^96^ | 2020 | Retrospective | 522 |  |  | 1 | 0 | 62 |  |  |  |  |
|  | Tohnosu et al^97^ | 1998 | Prospective | 50 |  | 200 | 1 | 5 | 52.9 |  |  |  |  |
|  | Valdes et al ^98^ | 2007 | Prospective | 12 |  | 72 | 6 |  |  | 23 |  | 33.3 | 15 |
|  | Valdes et al^99^ | 2007 | Prospective | 30 |  | 68 | 5 |  |  |  |  |  | 15 |

Tech, technique; SR, Specimen Radiography; OPT, Optical Spectroscopy; CYT, Cytology; IOUS, Intraoperative Ultrasound; FS, Frozen Section; MP, Margin Probe; MCT, Micro Computerised Topography; Pt, number of patients; Res, number of resections/specimens; Mar, number of margins; Ind, indication (1: BCS for BC; 2: BCS for impalpable BC; 3: BCS or mastectomy for BC; 4: BCS for DCIS; 5: Re-excision of BC after positive margins; 6: BCS for ILC); M Dist, positive margin distance in mm; CRR, cavity re-excision rate; PMR, positive margin rate; ROR, re-operation rate.

Table 2. Raw diagnostic accuracy data of studies included in meta-analysis

| Tech | Author | TP | FP | TN | FN | Total | Sensitivity | Specificity | PPV | NPV | Accuracy |
| --- | --- | --- | --- | --- | --- | --- | --- | --- | --- | --- | --- |
| SR | Lin et al^45^ | 24 | 10 | 158 | 13 | 205 | 64.9 | 94.1 | 70.6 | 92.4 | 88.8 |
|  | Bathla et al^46^ | 24 | 5 | 56 | 102 | 187 | 58.5 | 91.8 | 82.8 | 76.7 | 78.4 |
|  | Baù et al^47^ | 2 | 0 | 15 | 1 | 18 | 66.7 | 100 | 100 | 93.8 | 94.4 |
|  | Chagpar et al^48^ | 12 | 12 | 44 | 90 | 158 | 41.2 | 78.6 | 53.9 | 68.8 | 64.4 |
|  | Chand et al^49^ | 4 | 2 | 22 | 2 | 30 | 66.7 | 91.7 | 66.7 | 91.7 | 86.7 |
|  | Ciccarelli et al^50^ | 25 | 9 | 55 | 102 | 191 | 65.8 | 85.9 | 73.5 | 80.9 | 41.8 |
|  | Coombs et al^51^ | 12 | 4 | 25 | 52 | 93 | 52.2 | 86.2 | 75 | 69.4 | 39.8 |
|  | Funk et al^52^ | 114 | 331 | 2179 | 196 | 2820 | 36.8 | 86.8 | 25.6 | 91.8 | 81.3 |
|  | Graham et al^53^ | 62 | 1 | 18 | 119 | 200 | 62 | 95 | 98 | 32 | 67.2 |
|  | Hisada et al^54^ | 6 | 6 | 106 | 23 | 141 | 20.7 | 94.6 | 50 | 82.2 | 79.4 |
|  | Kulkarni et al^55^ | 23 | 123 | 538 | 24 | 708 | 48.9 | 81.4 | 15.8 | 95.7 | 79.2 |
|  | McCormick et al^56^ | 6 | 10 | 72 | 93 | 181 | 54.6 | 87.8 | 37.5 | 93.5 | 83.9 |
|  | Miller et al^57^ | 2 | 2 | 16 | 2 | 22 | 50 | 88.9 | 50 | 88.9 | 81.8 |
|  | Park et al^58^ | 14 | 61 | 24 | 0 | 99 | 100 | 28.2 | 18.7 | 100 | 38.4 |
|  | Pop et al^12^ | 4 | 11 | 63 | 5 | 83 | 44.4 | 85.1 | 26.7 | 92.7 | 80.7 |
|  | Prueksadee et al ^59^ | 3 | 3 | 1 | 12 | 19 | 37.5 | 25 | 50 | 16.7 | 33.3 |
|  | Saarela et al^60^ | 9 | 8 | 31 | 66 | 114 | 33 | 79 | 53 | 63 | 61 |
|  | Schaefgen et al^61^ | 13 | 62 | 87 | 12 | 174 | 52 | 58.4 | 17.3 | 87.9 | 57.5 |
|  | Stachs et al^62^ | 34 | 16 | 67 | 0 | 117 | 70 | 56.7 | 54.7 | 71.7 | 62.4 |
|  | Weber et al^13^ | 12 | 6 | 9 | 35 | 62 | 60 | 60 | 66.7 | 52.9 | 60 |
| OPT | Brown et al^63^ | 27 | 7 | 14 | 7 | 55 | 79.4 | 66.7 | 79.4 | 66.7 | 74.6 |
| OPT  OPT  OPT  OPT | Keller et al^64^ | 29 | 6 | 139 | 5 | 179 | 85.3 | 95.9 | 82.9 | 96.5 | 94 |
|  | Nguyen et al^65^ | 9 | 2 | 9 | 0 | 20 | 100 | 81.8 | 81.8 | 100 | 90 |
|  | Schmidt et al^66^ | 11 | 1 | 32 | 6 | 50 | 64.7 | 97 | 91.7 | 84.2 | 86 |
|  | Zhu et al^67^ | 222 | 26 | 454 | 18 | 720 | 92.5 | 94.6 | 89.5 | 96.2 | 93.9 |
| MP | Hoffman et al^68^ | 3 | 97 | 192 | 10 | 302 | 23.1 | 66.4 | 3 | 95.1 | 64.6 |
| MP  MP | Karni et al^69^ | 30 | 88 | 184 | 12 | 314 | 71.4 | 67.7 | 25.4 | 93.9 | 68.2 |
|  | LeeVan et al^70^ | 17 | 32 | 10 | 1 | 60 | 94.4 | 23.8 | 34.7 | 90.9 | 45 |
| MCT | McClatchy et al ^71^ | 3 | 9 | 18 | 2 | 32 | 60 | 66.7 | 25 | 90 | 65.6 |
| MCT  MCT | Qiu et al^35^ | 5 | 0 | 20 | 4 | 29 | 55.6 | 100 | 100 | 83.3 | 86.2 |
|  | Tang et al^36^ | 5 | 1 | 18 | 1 | 25 | 83.3 | 94.7 | 83.3 | 94.7 | 92 |
| IOUS | Kumar et al^15^ | 16 | 0 | 46 | 0 | 62 | 100 | 100 | 100 | 100 | 100 |
| IOUS  IOUS  IOUS  IOUS  IOUS  IOUS | Londero et al^72^ | 8 | 24 | 132 | 20 | 184 | 28.6 | 84.6 | 25 | 86.8 | 76.1 |
|  | Mesurolle et al ^73^ | 30 | 8 | 33 | 10 | 81 | 75 | 80.5 | 79 | 76.7 | 77.8 |
|  | Moschetta et al^74^ | 16 | 6 | 90 | 20 | 132 | 44.4 | 93.8 | 72.7 | 81.8 | 80.3 |
|  | Perera et al^75^ | 5 | 26 | 349 | 4 | 384 | 55.6 | 93.1 | 16.1 | 98.9 | 92.2 |
|  | Pop et al^12^ | 8 | 25 | 49 | 1 | 83 | 88.9 | 66.2 | 24.2 | 98 | 68.7 |
|  | Ramos et al^76^ | 24 | 79 | 116 | 6 | 225 | 80 | 59.5 | 95.1 | 23.3 | 62.2 |
| FS | Caruso et al^77^ | 5 | 3 | 44 | 1 | 53 | 83 | 93 | 62 | 97 | 94 |
| FS  FS  FS  FS  FS  FS  FS  FS  FS  FS  FS  FS  FS | Ikeda et al ^78^ | 17 | 4 | 34 | 1 | 56 | 94.4 | 89.5 | 81 | 97.1 | 91.1 |
|  | Jorns et al^79^ | 12 | 0 | 28 | 6 | 46 | 66.7 | 100 | 100 | 82.4 | 87 |
|  | Kikuyama et al^80^ | 287 | 18 | 440 | 18 | 763 | 94.1 | 96.1 | 94.1 | 96.1 | 95.3 |
|  | Kim et al^81^ | 3 | 1 | 23 | 2 | 29 | 60 | 95.8 | 75 | 92 | 89.7 |
|  | Ko et al^82^ | 120 | 1 | 338 | 24 | 483 | 83.3 | 99.7 | 99.2 | 93.4 | 94.8 |
|  | Kumar et al^15^ | 10 | 0 | 46 | 6 | 62 | 62.5 | 100 | 100 | 88.5 | 90.3 |
|  | Mahadevappa et al^14^ | 33 | 1 | 28 | 0 | 62 | 100 | 96.6 | 97.1 | 100 | 98.4 |
|  | Noguchi et al^83^ | 23 | 12 | 64 | 1 | 100 | 95.8 | 84.2 | 65.7 | 98.5 | 87 |
|  | Nowikiewicz et al^84^ | 4 | 0 | 429 | 72 | 505 | 5.3 | 100 | 100 | 85.6 | 85.7 |
|  | Olson et al^85^ | 57 | 5 | 1228 | 21 | 1311 | 73.1 | 99.6 | 91.9 | 98.3 | 98 |
|  | Osako et al^86^ | 259 | 53 | 955 | 60 | 1327 | 81.2 | 94.7 | 83 | 94.1 | 91.5 |
|  | Rusby et al^87^ | 39 | 15 | 495 | 8 | 557 | 83 | 97 | 72.2 | 98.4 | 96 |
|  | Weber et al^13^ | 32 | 5 | 35 | 8 | 80 | 80 | 87.5 | 86.5 | 81.4 | 83.8 |
| CYT | Bakhshandeh et al ^88^ | 30 | 7 | 472 | 1 | 510 | 97 | 99 | 81.1 | 99.8 | 98.4 |
| CYT  CYT  CYT  CYT  CYT  CYT  CYT  CYT  CYT  CYT  CYT  CYT | Blair et al^89^ | 3 | 0 | 115 | 1 | 119 | 75 | 100 | 100 | 99.1 | 99.2 |
|  | Cox et al^90^ | 22 | 3 | 86 | 0 | 111 | 100 | 96.6 | 88 | 100 | 97.3 |
|  | Creager et al ^91^ | 12 | 18 | 104 | 3 | 137 | 80 | 85.3 | 40 | 97.2 | 85 |
|  | D'Halluin et al^92^ | 71 | 26 | 304 | 9 | 410 | 88.6 | 92.2 | 73.6 | 97 | 91.5 |
|  | Ku et al^93^ | 17 | 2 | 68 | 0 | 87 | 100 | 97.1 | 89.5 | 100 | 97.7 |
|  | Mahadevappa et al ^14^ | 33 | 1 | 27 | 0 | 61 | 100 | 96.4 | 97.1 | 100 | 98.4 |
|  | Muttalib et al^94^ | 6 | 6 | 15 | 0 | 27 | 100 | 71.4 | 50 | 100 | 77.8 |
|  | Sumiyoshi et al^95^ | 14 | 4 | 136 | 6 | 160 | 70 | 97.1 | 77.8 | 95.8 | 93.8 |
|  | Tamanuki et al^96^ | 78 | 58 | 375 | 11 | 522 | 87.6 | 86.6 | 57.4 | 97.2 | 86.8 |
|  | Tohnosu et al^97^ | 27 | 16 | 156 | 1 | 200 | 96.4 | 90.7 | 62.8 | 99.4 | 91.5 |
|  | Valdes et al^98^ | 1 | 1 | 59 | 11 | 72 | 8.3 | 98.3 | 50 | 84.3 | 83.3 |
|  | Valdes et al^99^ | 3 | 11 | 53 | 1 | 68 | 75 | 82.8 | 21.4 | 98.2 | 82.4 |

Tech, technique; SR, Specimen Radiography; OPT, Optical Spectroscopy; CYT, Cytology; IOUS, Intraoperative Ultrasound; FS, Frozen Section; MP, Margin Probe; MCT, Micro Computerised Topography; TP, true positive; FP, false positive; TN, true negative; FN, false negative; PPV, positive predictive value; NPV, negative predictive value; Accuracy, diagnostic accuracy

Table 3. Meta-analysis: Summary estimates of sensitivity and specificity for all included studies for each IOMA technique type.

| Technique | No. of studies (patients/margins) | Sensitivity  (95% CI) | Variance Logit Sensitivity  (95% CI) | Specificity  (95% CI) | Variance Logit Specificity  (95% CI) |
| --- | --- | --- | --- | --- | --- |
| SR | 20 (5622) | 0.39 (0.24-0.56) | 2.32 (1.02-5.27) | 0.84 (0.77-0.89) | 0.89 (0.40-1.94) |
| OPT | 5 (1024) | 0.86 (0.76-0.93) | 0.34 (0.04-3.13) | 0.92 (0.82-0.97) | 0.78 (0.13-4.71) |
| CYT | 13 (2484) | 0.92 (0.77-0.98) | 3.68 (1.11-12.26) | 0.95 (0.90-0.97) | 1.21 (0.43-3.42) |
| IOUS | 7 (1151) | 0.72 (0.47-0.88) | 1.58 (0.33-7.62) | 0.87 (0.73-0.95) | 1.36 (0.31-5.87) |
| FS | 14 (5434) | 0.82 (0.66-0.91) | 2.30 (0.96-5.54) | 0.98 (0.95-0.99) | 2.84 (0.91-8.80) |
| MP* | 3 (165) | 0.73 (0.26-0.95) | 2.68 | 0.53 (0.30-0.75) | 0.67 |
| MCT* | 3 (68) | 0.65 (0.42-0.83) | 0 | 0.93 (0.56-0.99) | 2.52 |

*Only three studies and thus results should be interpreted with caution

Figure 1. PRISMA flow diagram

Figure 2. Pooled meta-analysis forest plot for each IOMA technique, displaying sensitivity and specificity data for all studies included and the pooled estimate.

**References**

1. Alkabban FM, Ferguson T. Breast Cancer. StatPearls. Treasure Island (FL): StatPearls Publishing

Copyright © 2022, StatPearls Publishing LLC.; 2022.

2. Nelson JA, Rubenstein RN, Haglich K, et al. Analysis of a Trend Reversal in US Lumpectomy Rates From 2005 Through 2017 Using 3 Nationwide Data Sets. JAMA Surgery 2022;157:702-11.

3. Jeevan R, Cromwell DA, Trivella M, et al. Reoperation rates after breast conserving surgery for breast cancer among women in England: retrospective study of hospital episode statistics. Bmj 2012;345:e4505.

4. Chakedis JM, Tang A, Savitz A, et al. Economic Impact of Reducing Reexcision Rates after Breast-Conserving Surgery in a Large, Integrated Health System. Ann Surg Oncol 2022;29:6288-96.

5. Kuritzky A, Reyna C, McGuire KP, et al. Evaluation of 2014 margin guidelines on re-excision and recurrence rates after breast conserving surgery: A multi-institution retrospective study. Breast 2020;51:29-33.

6. Bundred JR, Michael S, Stuart B, et al. Margin status and survival outcomes after breast cancer conservation surgery: prospectively registered systematic review and meta-analysis. Bmj 2022;378:e070346.

7. Moran MS, Schnitt SJ, Giuliano AE, et al. Society of Surgical Oncology-American Society for Radiation Oncology consensus guideline on margins for breast-conserving surgery with whole-breast irradiation in stages I and II invasive breast cancer. J Clin Oncol 2014;32:1507-15.

8. QUADAS-2: A Revised Tool for the Quality Assessment of Diagnostic Accuracy Studies. Annals of Internal Medicine 2011;155:529-36.

9. Harbord R. METANDI: Stata module to perform meta-analysis of diagnostic accuracy. 2008.

10. Nyaga VN, Arbyn M. Metadta: a Stata command for meta-analysis and meta-regression of diagnostic test accuracy data – a tutorial. Archives of Public Health 2022;80:95.

11. Reitsma JB, Glas AS, Rutjes AW, Scholten RJ, Bossuyt PM, Zwinderman AH. Bivariate analysis of sensitivity and specificity produces informative summary measures in diagnostic reviews. J Clin Epidemiol 2005;58:982-90.

12. Pop MM, Cristian S, Hanko-Bauer O, Ghiga DV, Georgescu R. Obtaining adequate surgical margin status in breast-conservation therapy: Intraoperative ultrasound-guided resection versus specimen mammography. Clujul Medical 2018;91:197-202.

13. Weber WP, Engelberger S, Viehl CT, et al. Accuracy of frozen section analysis versus specimen radiography during breast-conserving surgery for nonpalpable lesions. World J Surg 2008;32:2599-606.

14. Mahadevappa A, Nisha TG, Manjunath GV. Intra-operative Diagnosis of Breast Lesions by Imprint Cytology and Frozen Section with Histopathological Correlation. J Clin Diagn Res 2017;11:Ec01-ec6.

15. Kumar N, on M, Chintamani C. Intraoperative Specimen Ultrasonography: Is It a Reliable Tool for Margin Assessment Following Breast Conservation Surgery for Breast Carcinoma? Cureus 2021;13:e15806.

16. Hassan Ali S, SP S, N AK. Rate of breast-conserving surgery vs mastectomy in breast cancer: a tertiary care centre experience from South India. Indian Journal of Surgical Oncology 2019;10:72-6.

17. Crown A, Handy N, Weed C, Laskin R, Rocha FG, Grumley J. Oncoplastic Breast-Conserving Surgery: Can We Reduce Rates of Mastectomy and Chemotherapy Use in Patients with Traditional Indications for Mastectomy? Ann Surg Oncol 2021;28:2199-209.

18. Schulman AM, Mirrielees JA, Leverson G, Landercasper J, Greenberg C, Wilke LG. Reexcision surgery for breast cancer: an analysis of the American Society of Breast Surgeons (ASBrS) Mastery SM database following the SSO-ASTRO “no ink on tumor” guidelines. Annals of surgical oncology 2017;24:52-8.

19. Tang SS-K, Kaptanis S, Haddow JB, et al. Current margin practice and effect on re-excision rates following the publication of the SSO-ASTRO consensus and ABS consensus guidelines: a national prospective study of 2858 women undergoing breast-conserving therapy in the UK and Ireland. European Journal of Cancer 2017;84:315-24.

20. Mamtani A, Zabor EC, Rosenberger LH, Stempel M, Gemignani ML, Morrow M. Was reexcision less frequent for patients with lobular breast cancer after publication of the SSO-ASTRO margin guidelines? Annals of surgical oncology 2019;26:3856-62.

21. Pleijhuis RG, Graafland M, de Vries J, Bart J, de Jong JS, van Dam GM. Obtaining adequate surgical margins in breast-conserving therapy for patients with early-stage breast cancer: current modalities and future directions. Annals of surgical oncology 2009;16:2717-30.

22. Menes TS, Tartter PI, Bleiweiss I, Godbold JH, Estabrook A, Smith SR. The consequence of multiple re-excisions to obtain clear lumpectomy margins in breast cancer patients. Annals of surgical oncology 2005;12:881-5.

23. Kouzminova NB, Aggarwal S, Aggarwal A, Allo MD, Lin AY. Impact of initial surgical margins and residual cancer upon re-excision on outcome of patients with localized breast cancer. The American journal of surgery 2009;198:771-80.

24. Garcia MT, Mota BS, Cardoso N, et al. Accuracy of frozen section in intraoperative margin assessment for breast-conserving surgery: A systematic review and meta-analysis. PLoS One 2021;16:e0248768.

25. Uecker JM, Bui EH, Foulkrod KH, Sabra JP. Intraoperative assessment of breast cancer specimens decreases cost and number of reoperations. The American surgeon 2011;77:342-4.

26. Buchholz TA, Somerfield MR, Griggs JJ, et al. Margins for breast-conserving surgery with whole-breast irradiation in stage I and II invasive breast cancer: American Society of Clinical Oncology endorsement of the Society of Surgical Oncology/American Society for Radiation Oncology consensus guideline. J Clin Oncol 2014;32:1502-6.

27. Morrow M, Van Zee KJ, Solin LJ, et al. Society of Surgical Oncology-American Society for Radiation Oncology-American Society of Clinical Oncology Consensus Guideline on Margins for Breast-Conserving Surgery with Whole-Breast Irradiation in Ductal Carcinoma In Situ. Ann Surg Oncol 2016;23:3801-10.

28. Shah C, Hobbs BP, Vicini F, et al. The Diminishing Impact of Margin Definitions and Width on Local Recurrence Rates following Breast-Conserving Therapy for Early-Stage Invasive Cancer: A Meta-Analysis. Ann Surg Oncol 2020;27:4628-36.

29. Marinovich ML, Azizi L, Macaskill P, et al. The Association of Surgical Margins and Local Recurrence in Women with Ductal Carcinoma In Situ Treated with Breast-Conserving Therapy: A Meta-Analysis. Ann Surg Oncol 2016;23:3811-21.

30. Esbona K, Li Z, Wilke LG. Intraoperative imprint cytology and frozen section pathology for margin assessment in breast conservation surgery: a systematic review. Annals of surgical oncology 2012;19:3236-45.

31. Boichenko E, Kirsanov D. Optical spectroscopy and chemometrics in intraoperative tumor margin assessment. TrAC Trends in Analytical Chemistry 2023:116955.

32. Zúñiga WC, Jones V, Anderson SM, et al. Raman spectroscopy for rapid evaluation of surgical margins during breast cancer lumpectomy. Scientific reports 2019;9:14639.

33. Wide Field OCT + AI for Positive Margin Rates in Breast Conservation Surgery. at <https://classic.clinicaltrials.gov/show/NCT05113927>.)

34. Thill M, Dittmer C, Baumann K, Friedrichs K, Blohmer JU. MarginProbe®--final results of the German post-market study in breast conserving surgery of ductal carcinoma in situ. Breast 2014;23:94-6.

35. Qiu SQ, Dorrius MD, de Jongh SJ, et al. Micro-computed tomography (micro-CT) for intraoperative surgical margin assessment of breast cancer: A feasibility study in breast conserving surgery. Eur J Surg Oncol 2018;44:1708-13.

36. Tang R, Coopey SB, Buckley JM, et al. A pilot study evaluating shaved cavity margins with micro-computed tomography: A novel method for predicting lumpectomy margin status intraoperatively. Breast Journal 2013;19:485-9.

37. Thill M, Szwarcfiter I, Kelling K, et al. Magnetic resonance imaging system for intraoperative margin assessment for DCIS and invasive breast cancer using the ClearSight™ system in breast-conserving surgery—Results from a postmarketing study. Journal of Surgical Oncology 2022;125:361-8.

38. Papa M, Allweis T, Karni T, et al. An intraoperative MRI system for margin assessment in breast conserving surgery: Initial results from a novel technique. J Surg Oncol 2016;114:22-6.

39. Miripour ZS, Abbasv, i F, et al. Human study on cancer diagnostic probe (CDP) for real-time excising of breast positive cavity side margins based on tracing hypoxia glycolysis; checking diagnostic accuracy in non-neoadjuvant cases. Cancer Medicine 2022;11:1630-45.

40. Kubo A, Tanei T, A RP, et al. Comparison of "click-to-sense" assay with frozen section analysis using simulated surgical margins in breast cancer patients. Eur J Surg Oncol 2022;48:1520-6.

41. Brachtel EF, Johnson NB, Huck AE, et al. Spectrally encoded confocal microscopy for diagnosing breast cancer in excision and margin specimens. Laboratory Investigation 2016;96:459-67.

42. Chang TP, Leff DR, Shousha S, et al. Imaging breast cancer morphology using probe-based confocal laser endomicroscopy: towards a real-time intraoperative imaging tool for cavity scanning. Breast Cancer Res Treat 2015;153:299-310.

43. St John ER, Balog J, McKenzie JS, et al. Rapid evaporative ionisation mass spectrometry of electrosurgical vapours for the identification of breast pathology: towards an intelligent knife for breast cancer surgery. Breast Cancer Res 2017;19:59.

44. Real Time Tissue Characterisation Using Mass Spectrometry REI-EXCISE iKnife Study. at <https://classic.clinicaltrials.gov/show/NCT03432429>.)

45. Lin C, Wang KY, Xu F, et al. The application of intraoperative specimen mammography for margin status assessment in breast-conserving surgery: A single-center retrospective study. Breast Journal 2020;26:1871-3.

46. Bathla L, Harris A, Davey M, Sharma P, Silva E. High resolution intra-operative two-dimensional specimen mammography and its impact on second operation for re-excision of positive margins at final pathology after breast conservation surgery. American Journal of Surgery 2011;202:387-94.

47. Baù MG, Surace A, Gregori G, et al. Vacuum intraoperative specimen mammography: A novel technique. European Journal of Obstetrics and Gynecology and Reproductive Biology 2020;253:1-6.

48. Chagpar AB, Butler M, Killelea BK, Horowitz NR, Stavris K, Lannin DR. Does three-dimensional intraoperative specimen imaging reduce the need for re-excision in breast cancer patients? A prospective cohort study. American Journal of Surgery 2015;210:886-90.

49. Ch, JT, Sharma MM, Dharmarajan JP, Nambiar A. Digital Breast Tomosynthesis as a Tool in Confirming Negative Surgical Margins in Non-palpable Breast Lesions. Indian Journal of Surgical Oncology 2019;10:624-8.

50. Ciccarelli G, Di Virgilio MR, Menna S, et al. Radiography of the surgical specimen in early stage breast lesions: diagnostic reliability in the analysis of the resection margins. Radiol Med 2007;112:366-76.

51. Coombs NJ, Vassallo PP, Parker AJ, Yiangou C. Radiological review of specimen radiographs after breast localisation biopsy is not always necessary. Eur J Surg Oncol 2006;32:516-9.

52. Funk A, Heil J, Harcos A, et al. Efficacy of intraoperative specimen radiography as margin assessment tool in breast conserving surgery. Breast Cancer Research and Treatment 2020;179:425-33.

53. RA G, MJ H, CJ S, et al. The efficacy of specimen radiography in evaluating the surgical margins of impalpable breast carcinoma. AJR American journal of roentgenology 1994;162:33-6.

54. Hisada T, Sawaki M, Ishiguro J, et al. Impact of intraoperative specimen mammography on margins in breast-conserving surgery. Mol Clin Oncol 2016;5:269-72.

55. Kulkarni SA, Kulkarni K, Schacht D, et al. High-Resolution Full-3D Specimen Imaging for Lumpectomy Margin Assessment in Breast Cancer. Ann Surg Oncol 2021;28:5513-24.

56. McCormick JT, Keleher AJ, Tikhomirov VB, Budway RJ, Caushaj PF. Analysis of the use of specimen mammography in breast conservation therapy. Am J Surg 2004;188:433-6.

57. Miller CL, Coopey SB, Rafferty E, Gadd M, Smith BL, Specht MC. Comparison of intra-operative specimen mammography to standard specimen mammography for excision of non-palpable breast lesions: a randomized trial. Breast Cancer Res Treat 2016;155:513-9.

58. Park KU, Kuerer HM, Rauch GM, et al. Digital Breast Tomosynthesis for Intraoperative Margin Assessment during Breast-Conserving Surgery. Ann Surg Oncol 2019;26:1720-8.

59. Prueksadee J, Khamapirad T. Margin determination of two-view specimen radiography in breast cancer. Asian Biomedicine 2009;3:537-43.

60. AO S, TJ R, KM L, et al. Wire-guided excision of non-palpable breast cancer: determinants and correlations between radiologic and histologic margins and residual disease in re-excisions. Breast (Edinburgh, Scotland) 2001;10:28-34.

61. Schaefgen B, Funk A, Sinn HP, et al. Does conventional specimen radiography after neoadjuvant chemotherapy of breast cancer help to reduce the rate of second surgeries? Breast Cancer Res Treat 2022;191:589-98.

62. Stachs A, Bollmann J, Martin A, et al. Radiopaque tissue transfer and X-ray system versus standard specimen radiography for intraoperative margin assessment in breast-conserving surgery: randomized clinical trial. BJS Open 2022;6.

63. Brown JQ, Bydlon TM, Richards LM, et al. Optical assessment of tumor resection margins in the breast. IEEE J Sel Top Quantum Electron 2010;16:530-44.

64. Keller MD, Majumder SK, Kelley MC, et al. Autofluorescence and diffuse reflectance spectroscopy and spectral imaging for breast surgical margin analysis. Lasers Surg Med 2010;42:15-23.

65. Nguyen FT, Zysk AM, Chaney EJ, et al. Intraoperative evaluation of breast tumor margins with optical coherence tomography. Cancer Res 2009;69:8790-6.

66. Schmidt H, Connolly C, Jaffer S, et al. Evaluation of surgically excised breast tissue microstructure using wide-field optical coherence tomography. Breast J 2020;26:917-23.

67. Zhu D, Wang J, Marjanovic M, et al. Differentiation of breast tissue types for surgical margin assessment using machine learning and polarization-sensitive optical coherence tomography. Biomedical Optics Express 2021;12:3021-36.

68. Hoffman A, Ashkenazi I. The efficiency of MarginProbe in detecting positive resection margins in epithelial breast cancer following breast conserving surgery. European Journal of Surgical Oncology 2022;48:1498-502.

69. Karni T, Pappo I, bank J, et al. A device for real-time, intraoperative margin assessment in breast-conservation surgery. American Journal of Surgery 2007;194:467-73.

70. LeeVan E, Ho BT, Seto S, Shen J. Use of MarginProbe as an adjunct to standard operating procedure does not significantly reduce re-excision rates in breast conserving surgery. Breast Cancer Res Treat 2020;183:145-51.

71. McClatchy DM, 3rd, Zuurbier RA, Wells WA, Paulsen KD, Pogue BW. Micro-computed tomography enables rapid surgical margin assessment during breast conserving surgery (BCS): correlation of whole BCS micro-CT readings to final histopathology. Breast Cancer Res Treat 2018;172:587-95.

72. V L, C Z, M P, A L, R G, M B. Surgical specimen ultrasound: is it able to predict the status of resection margins after breast-conserving surgery? Breast (Edinburgh, Scotland) 2010;19:532-7.

73. B M, M E-K, D H, et al. Sonography of postexcision specimens of nonpalpable breast lesions: value, limitations, and description of a method. AJR American journal of roentgenology 2006;186:1014-24.

74. M M, M T, T I, et al. Role of specimen US for predicting resection margin status in breast conserving therapy2015.

75. Perera N, Bourke AG. The technique and accuracy of breast specimen ultrasound in achieving clear margins in breast conserving surgery. Journal of Medical Imaging and Radiation Oncology 2020;64:747-55.

76. Ramos M, Díaz JC, Ramos T, et al. Ultrasound-guided excision combined with intraoperative assessment of gross macroscopic margins decreases the rate of reoperations for non-palpable invasive breast cancer. Breast 2013;22:520-4.

77. Caruso F, Ferrara M, Castiglione G, et al. Therapeutic mammaplasties: full local control of breast cancer in one surgical stage with frozen section. Eur J Surg Oncol 2011;37:871-5.

78. Ikeda T, Enomoto K, Wada K, et al. Frozen-section-guided breast-conserving surgery: Implications of diagnosis by frozen section as a guide to determining the extent of resection. Surgery Today 1997;27:207-12.

79. Jorns JM, Daignault S, Sabel MS, Wu AJ. Is intraoperative frozen section analysis of Reexcision specimens of value in preventing Reoperation in breast-conserving therapy? American Journal of Clinical Pathology 2014;142:601-8.

80. Kikuyama M, Akashi-Tanaka S, Hojo T, et al. Utility of intraoperative frozen section examinations of surgical margins: implication of margin-exposed tumor component features on further surgical treatment. Jpn J Clin Oncol 2015;45:19-25.

81. Kim MJ, Kim CS, Park YS, Choi EH, Han KD. The efficacy of intraoperative frozen section analysis during breast-conserving surgery for patients with ductal carcinoma in situ. Breast Cancer: Basic and Clinical Research 2016;10:205-10.

82. Ko S, Chun YK, Kang SS, Hur MH. The Usefulness of Intraoperative Circumferential Frozen-Section Analysis of Lumpectomy Margins in Breast-Conserving Surgery. J Breast Cancer 2017;20:176-82.

83. Noguchi M, Minami M, Earashi M, et al. Intraoperative histologic assessment of surgical margins and lymph node metastasis in breast-conserving surgery. J Surg Oncol 1995;60:185-90.

84. Nowikiewicz T, Śrutek E, Głowacka-Mrotek I, Tarkowska M, Żyromska A, Zegarski W. Clinical outcomes of an intraoperative surgical margin assessment using the fresh frozen section method in patients with invasive breast cancer undergoing breast-conserving surgery - a single center analysis. Scientific reports 2019;9:13441.

85. Olson TP, Harter J, Muñoz A, Mahvi DM, Breslin T. Frozen section analysis for intraoperative margin assessment during breast-conserving surgery results in low rates of re-excision and local recurrence. Ann Surg Oncol 2007;14:2953-60.

86. Osako T, Nishimura R, Nishiyama Y, et al. Efficacy of intraoperative entire-circumferential frozen section analysis of lumpectomy margins during breast-conserving surgery for breast cancer. Int J Clin Oncol 2015;20:1093-101.

87. Rusby JE, Paramanathan N, Laws SA, Rainsbury RM. Immediate latissimus dorsi miniflap volume replacement for partial mastectomy: use of intra-operative frozen sections to confirm negative margins. Am J Surg 2008;196:512-8.

88. Bakhsh, eh M, Tutuncuoglu SO, Fischer G, Masood S. Use of imprint cytology for assessment of surgical margins in lumpectomy specimens of breast cancer patients. Diagn Cytopathol 2007;35:656-9.

89. Blair SL, Wang-Rodriguez J, Cortes-Mateos MJ, et al. Enhanced touch preps improve the ease of interpretation of intraoperative breast cancer margins. Am Surg 2007;73:973-6.

90. Cox CE, Ku NN, Reintgen DS, Greenberg HM, Nicosia SV, Wangensteen S. Touch preparation cytology of breast lumpectomy margins with histologic correlation. Arch Surg 1991;126:490-3.

91. Creager AJ, Shaw JA, Young PR, Geisinger KR. Intraoperative evaluation of lumpectomy margins by imprint cytology with histologic correlation: A community hospital experience. Archives of Pathology and Laboratory Medicine 2002;126:846-8.

92. D'Halluin F, Tas P, Rouquette S, et al. Intra-operative touch preparation cytology following lumpectomy for breast cancer: a series of 400 procedures. Breast 2009;18:248-53.

93. Ku NN, Cox CE, Reintgen DS, Greenberg HM, Nicosia SV. Cytology of lumpectomy specimens. Acta Cytol 1991;35:417-21.

94. Muttalib M, Tisdall M, Scawn R, Shousha S, Cummins RS, Sinnett HD. Intra-operative specimen analysis using faxitron microradiography for excision of mammographically suspicious, non-palpable breast lesions. Breast 2004;13:307-15.

95. Sumiyoshi K, Nohara T, Iwamoto M, et al. Usefulness of intraoperative touch smear cytology in breast-conserving surgery. Exp Ther Med 2010;1:641-5.

96. Tamanuki T, Namura M, Aoyagi T, Shimizu S, Suwa T, Matsuzaki H. Effect of Intraoperative Imprint Cytology Followed by Frozen Section on Margin Assessment in Breast-Conserving Surgery. Ann Surg Oncol 2021;28:1338-46.

97. Tohnosu N, Nabeya Y, Matsuda M, et al. Rapid Intraoperative Scrape Cytology Assessment of Surgical Margins in Breast Conservation Surgery. Breast Cancer 1998;5:165-9.

98. Valdes EK, Boolbol SK, Ali I, Feldman SM, Cohen JM. Intraoperative touch preparation cytology for margin assessment in breast-conservation surgery: does it work for lobular carcinoma? Ann Surg Oncol 2007;14:2940-5.

99. Valdes EK, Boolbol SK, Cohen JM, Feldman SM. Intra-operative touch preparation cytology; does it have a role in re-excision lumpectomy? Annals of Surgical Oncology 2007;14:1045-50.

Supplementary Figure 1: Receiver operating characteristic graph with 95% confidence region and 95% prediction region for SR

Supplementary Figure 2: Receiver operating characteristic graph with 95% confidence region and 95% prediction region for OPT

Supplementary Figure 3: Receiver operating characteristic graph with 95% confidence region and 95% prediction region for CYT

Supplementary Figure 4: Receiver operating characteristic graph with 95% confidence region and 95% prediction region for IOUS

Supplementary Figure 5: Receiver operating characteristic graph with 95% confidence region and 95% prediction region for FS

Supplementary Figure 6: Receiver operating characteristic graph with 95% confidence region and 95% prediction region for MP

Supplementary Figure 7: Receiver operating characteristic graph with 95% confidence region and 95% prediction region for MCT

Supplementary Figure 8: Quality scoring for all studies


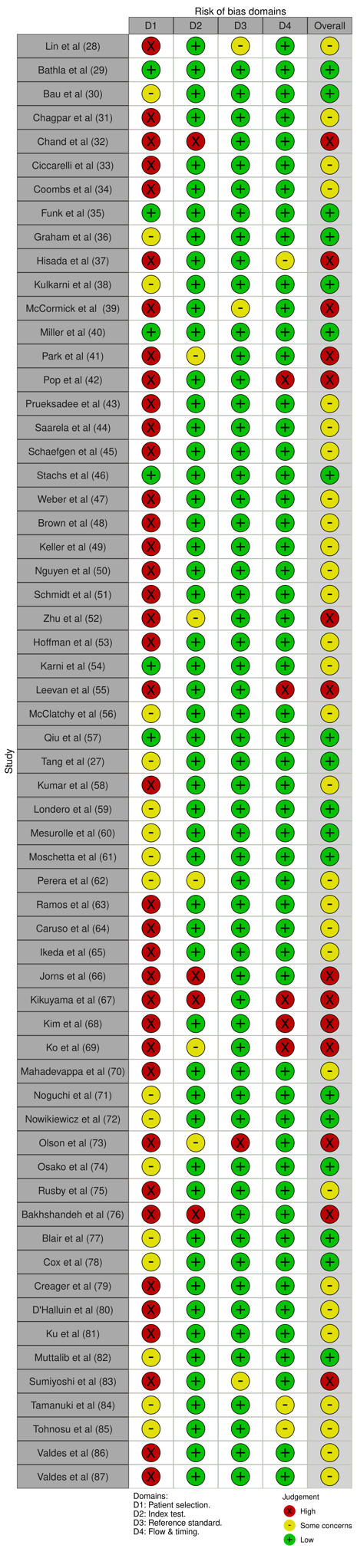


Supplementary Figure 9: Summary of quality scoring/ risk of bias for all studies


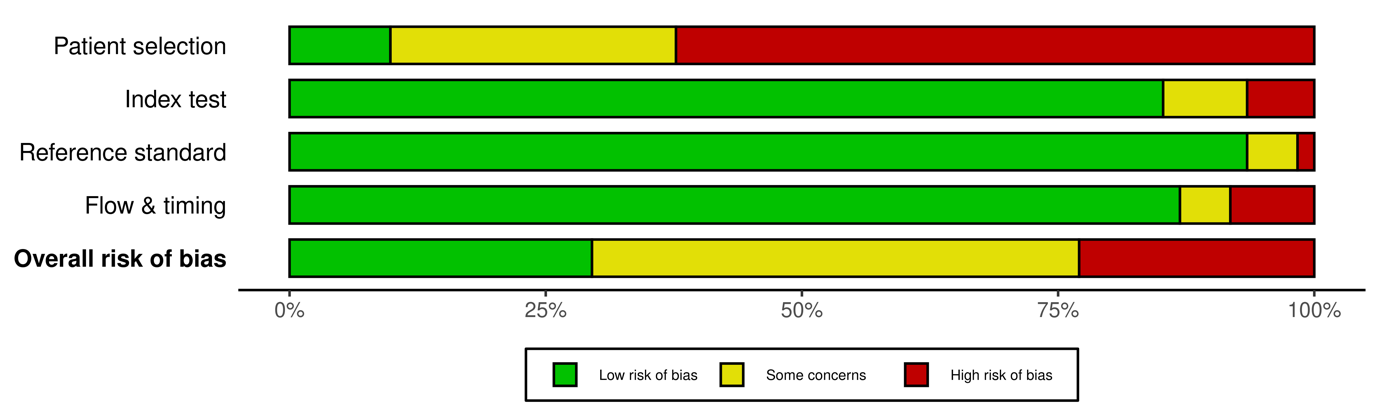

Supplement: Multimedia component 1 [file mmc1.docx]
